# Aerogel‐Based Biomaterials for Biomedical Applications: From Fabrication Methods to Disease‐Targeting Applications

**DOI:** 10.1002/advs.202204681

**Published:** 2023-05-22

**Authors:** Solmaz Karamikamkar, Ezgi Pinar Yalcintas, Reihaneh Haghniaz, Natan Roberto de Barros, Marvin Mecwan, Rohollah Nasiri, Elham Davoodi, Fatemeh Nasrollahi, Ahmet Erdem, Heemin Kang, Junmin Lee, Yangzhi Zhu, Samad Ahadian, Vadim Jucaud, Hajar Maleki, Mehmet Remzi Dokmeci, Han‐Jun Kim, Ali Khademhosseini

**Affiliations:** ^1^ Terasaki Institute for Biomedical Innovation (TIBI) Los Angeles CA 90024 USA; ^2^ Department of Mechanical and Mechatronics Engineering University of Waterloo Waterloo ON N2L 3G1 Canada; ^3^ Department of Bioengineering University of California‐Los Angeles (UCLA) Los Angeles CA 90095 USA; ^4^ Department of Biomedical Engineering Kocaeli University Umuttepe Campus Kocaeli 41001 Turkey; ^5^ Department of Materials Science and Engineering Korea University Seoul 02841 Republic of Korea; ^6^ Department of Materials Science and Engineering Pohang University of Science and Technology (POSTECH) Pohang 37673 Republic of Korea; ^7^ Institute of Inorganic Chemistry Department of Chemistry University of Cologne Greinstraße 6 50939 Cologne Germany; ^8^ Center for Molecular Medicine Cologne CMMC Research Center Robert‐Koch‐Str. 21 50931 Cologne Germany; ^9^ College of Pharmacy Korea University Sejong 30019 Republic of Korea

**Keywords:** additive manufacturing, aerogel, diagnosis, drug delivery, microfluidics, tissue engineering, wound healing

## Abstract

Aerogel‐based biomaterials are increasingly being considered for biomedical applications due to their unique properties such as high porosity, hierarchical porous network, and large specific pore surface area. Depending on the pore size of the aerogel, biological effects such as cell adhesion, fluid absorption, oxygen permeability, and metabolite exchange can be altered. Based on the diverse potential of aerogels in biomedical applications, this paper provides a comprehensive review of fabrication processes including sol‐gel, aging, drying, and self‐assembly along with the materials that can be used to form aerogels. In addition to the technology utilizing aerogel itself, it also provides insight into the applicability of aerogel based on additive manufacturing technology. To this end, how microfluidic‐based technologies and 3D printing can be combined with aerogel‐based materials for biomedical applications is discussed. Furthermore, previously reported examples of aerogels for regenerative medicine and biomedical applications are thoroughly reviewed. A wide range of applications with aerogels including wound healing, drug delivery, tissue engineering, and diagnostics are demonstrated. Finally, the prospects for aerogel‐based biomedical applications are presented. The understanding of the fabrication, modification, and applicability of aerogels through this study is expected to shed light on the biomedical utilization of aerogels.

## Graphical abstract

Aerogel‐based biomaterials are increasingly being considered for biomedical applications due to their unique properties such as high porosity, hierarchical porous network, and large specific pore surface area. Based on the diverse potential of aerogels, this paper provides a comprehensive review of fabrication processes, materials, and additive manufacturing along with the biomedical application utilizing aerogel technology.

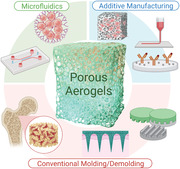

## 1 Introduction

Aerogels are a group of materials, composed of organic and inorganic building blocks with the unique properties of very low density, high porosity, open pores, and large pore surface area.^[^ ^1^ ^]^ The porous structure of the aerogel is filled with air inside and consists of a highly crosslinked 3D solid network. In general, aerogels are prepared through the drying process of wet gels (e.g., alcohol gels, hydrogels), and can be used for applications requiring liquid adsorption based on the properties of “dried solid materials”. However, even if gels are dried, not all dried gels have aerogel properties. In general, porous dry gels can be divided into xerogels and aerogels according to the characteristics of their porous structure under drying conditions.^[^ ^2^ ^]^

Among the porous dry gel types, aerogels are the most uniform porous materials obtained by suppressing shrinkage during drying, unlike xerogel, which shrinks significantly upon drying. Although xerogels mostly contain nanoscale pores that might be suitable for breathable materials, they lack a homogeneous pore distribution and may have defects throughout the solid network due to shrinkage. However, aerogel fabrication via a controlled drying process can produce solid porous materials with uniformly distributed pores at both the nano‐ and micro‐scales. Therefore, the advancement and development of highly controlled fabrication processes to maintain these unique properties and functions of aerogels have been studied for decades.^[^ ^3^ ^]^

Aerogels can often be confused with foam materials. However, there are distinct differences between the two materials in the fabrication process. The foam materials are processed through gas dissolution in a polymer melt and later release gas during polymer solidification to create porous solid materials with micron‐scale pores. This process usually results in the production of porous material with either open or closed pores called foams. The final porous material whether having open or closed pores does not provide interconnected pores as the pores are the result of removed nucleated cells. Aerogel materials, however, undergo a supercritical drying (SCD) process in which a liquid trapped in a gel network turns into a supercritical state and later becomes a gas, leaving a structure of a porous solid material. This process preserves the pores created during sol‐gel conversion at the nano‐to‐micron scale, allowing for the interconnection of pores throughout the aerogel network. This is due to the fact that the second phase is a liquid throughout the whole material and its removal will leave continuous connected pores throughout the whole structure. The properties of the aerogel can also be adjusted by controlling the interfaces between the components used in their synthesis.^[^ ^4^ ^]^ In particular, the hybridization of inorganic precursors with organic moieties can further improve mechanical strength, hydrophilicity or hydrophobicity, and chemical function.^[^ ^1^, ^4^ ^]^ These hybrid aerogels exhibit novel properties such as electrical and magnetic behavior, enhanced adsorption, and improved structural function, expanding their potential in regenerative medicine and biomedical applications.^[^ ^5^ ^]^ The manufacturing methods and materials that make up the aerogel are discussed with various examples in Section 2 and Section 3, respectively.

The application of aerogels for regenerative and biomedical purposes requires the precise preparation of the material in a form specific to that application. As one of these transformation strategies, a technique for fabricating high‐resolution scaffolds using sol‐gel chemistry with microfluidics or microfabrication techniques has been proposed. This procedure can also lead to aerogels with micron‐size porosity needed for cell encapsulation and infiltration. However, the process to make aerogels involves making gel materials followed by removing solvent (drying), resulting in a porous material with interconnected pores. This is due to the fact that the second phase is a liquid throughout the whole material and its removal will leave continuous connected pores throughout the whole structure.

The sol‐gel chemistry can be carried out inside the microfluidic channels through external or internal gelation by adding crosslinking agents.^[^ ^6^ ^]^ For example, the production of aerogels through droplet‐based microfluidics requires the generation of hydrogels in the microfluidic systems, followed by the drying process while maintaining the particles’ porous structure. This approach requires a precise fabrication design to prepare an aerogel or aerogel‐inspired material with hierarchal pore size assembly. In addition, 3D printing can be another promising approach for building high‐resolution aerogel structures. Aerogels by 3D printing methods with enlarged porosities and customized micromorphology had increased cell viability that enabled effective regenerative medicine approaches owing to their porous structure.^[^ ^7^ ^]^ The fabrication and application of aerogel‐based scaffolds using various additive manufacturing (AM) methods will be covered in detail in Section 4.

**Scheme 1:**
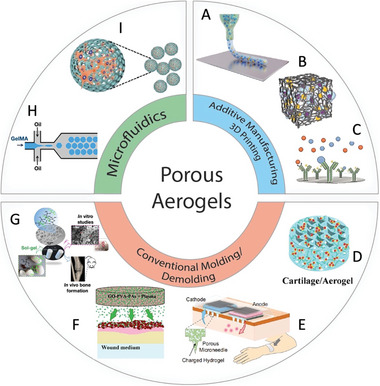
A,B) Aerogel scaffold fabrication using additive manufacturing. C) Aerogel as biosensors and diagnostics. D) Aerogel as a cell delivery scaffold. E) Aerogel Microneedles. Application of aerogels for (F) wound healing and (G) bone regeneration. H) Microfluidic chip. I) Aerogel as a cell microcarrier via microfluidic device. A) Reproduced with permission.^[^ ^12^ ^]^ Copyright 2020, Springer Nature. B) Reproduced with permission.^[^ ^13^ ^]^ Copyright 2020, De Gruyter. C) Reproduced with permission.^[^ ^14^ ^]^ Copyright 2021, MDPI. D) Reproduced with permission.^[^ ^15^ ^]^ Copyright 2020, MDPI. E) Reproduced with permission.^[^ ^16^ ^]^ Copyright 2021, Springer Nature. F) Reproduced with permission.^[^ ^17^ ^]^ Copyright 2018, American Chemical Society. G) Reproduced with permission.^[^ ^18^ ^]^ Copyright 2019, American Chemical Society. H) Reproduced with permission.^[^ ^19^ ^]^ Copyright 2019, American Chemical Society. I) Reproduced with permission.^[^ ^4b^ ^]^ Copyright 2018, American Chemical Society.

As mentioned above, aerogels with precisely engineered porosity have great potential for numerous applications in biomedical fields. For example, inherent properties of the aerogel‐based biomaterials, such as large surface area (>1000 m^2^ g^−1^), highly porous network (>95%), and exceptional permeability, make them suitable candidates for absorbing exudates in wound healing applications. Besides, aerogels are also suitable for drug delivery systems (DDS) because of their high porosity and increased surface area.^[^ ^8^ ^]^ It is worth to be mentioned that there are some reported low surface area aerogels which are due to the high shrinkage as well as a highly concentrated solution that can be avoided by optimization of the processing condition. The nano‐sized pores of the aerogel can not only load a large amount of drug with rapid water absorption but also act as a drug matrix that can accelerate drug release. These aerogel properties demonstrate the potential for effective use as drug carriers.^[^ ^9^ ^]^ Aerogels can be used as scaffolds for tissue engineering.^[^ ^10^ ^]^ The high extent of micro and mesoporosity in aerogels, together with additional macro size porosities developed through fabrication techniques (hierarchically organized pores), enhance oxygen permeability and improve the exchange of metabolites, which leads to superior cell activity, adhesion, and proliferation.^[^ ^10^, ^11^ ^]^ All these applications of novel aerogels are reviewed in this article (Section 5, **Scheme** **1**).

This review aims to summarize the latest findings with such interesting examples in relation to 1) fabrication methods, 2) materials, 3) AM, and 4) biomedical applications of aerogel‐based biomaterials. First, the fabrication mechanism based on the sol‐gel, aging, drying, and self‐assembly processes is described. Next, the characteristics of organic, inorganic, carbonous, and hybrid materials, which are materials that can make aerogels, are summarized and discussed. The macroscopic shape and microstructural design of aerogel scaffolds is presented through AM, including microfluidic engineering and 3D printing approaches. Finally, research in the realm of wound healing, drug delivery, tissue engineering, and diagnosis, together with their outlook, are explained. The understanding of the fabrication, modification, and applicability of aerogels through this study is expected to shed light on the biomedical uses of aerogels.

## 2 Fabrication Processes of Aerogel and Aerogel‐Inspired Materials

Fundamental steps involved in the aerogel preparations are gelation (sol‐gel), aging, drying, and post‐synthesis treatments.^[^ ^20^ ^]^ All these steps contribute to the gel structure, thus affecting its function and related applications. There are several comprehensive review papers that discuss these steps in detail.^[^ ^2^, ^21^ ^]^ A short summary of each step is summarized in this section.

### 2.1 Sol‐Gel

The first step of the sol‐gel process is to prepare a sol, which is a stable suspension of solid colloidal particles in a solvent. A colloidal suspension is created when solid nanosized particles generated from a precursor material are dispersed within a solvent. These precursors must be soluble in the solvent medium and sufficiently reactive to participate in the gel‐forming process.

In the sol preparation step of the sol‐gel process, the conversion of alkoxide precursors to colloidal suspensions occurs through two reactions: hydrolysis and condensation. During hydrolysis, alkoxides (‐OR) in the structure are (partially or completely) turned into hydroxyl groups (‐OH) due to the nucleophilic attack of water. In the condensation reaction step, water (oxidation) or alcohol (alkoxylation) is released between two Si‐OH species or between Si‐OH and Si‐OR, respectively. These reactions trigger the development of newly crosslinked Si—O—Si bonds.^[^ ^22^ ^]^ These hydrolysis and condensation reactions cause nucleation and growth to form an amorphous oxidic sol.^[^ ^23^ ^]^ The transition from the sol state to the gel state is achieved by crosslinking due to prolonged condensation. Although the chemistry of sol‐gel reaction is well known, the high rates of hydrolysis and condensation reactions are major issues that make the process difficult to control. This uncontrollable gelation behavior makes it difficult to tune the final porosity in conventional sol‐gel approaches (i.e., nucleation and growth).^[^ ^22^, ^24^ ^]^ It is also important not to terminate the gelation reaction of chemicals in aerogel formation to ensure structural change during aging. Si‐OH and unreacted Si‐OR remaining in the network after gelation and monomer trapped in the pores can be a significant problem in sol‐gel practice.

### 2.2 Self‐Assembly

While sol‐gel processing of molecular precursors is used to synthesize traditional metal oxide‐based aerogels, the self‐assembly approach is a potent technique for preparing biopolymeric gel network build‐up. With this approach, a number of biopolymeric systems (i.e., polysaccharides, polypeptides) can interact and assemble to form the 3D network through various mechanisms.^[^ ^25^ ^]^ Within the self‐assembly process, the macromolecular chains of biopolymers can interact through covalent (e.g., via crosslinking) and non‐covalent interactions (e.g., changing the pH of the solution to establish hydrogen bondings, the addition of surfactant and ions, temperature, etc.). The obtained 3D hydrogel network could undergo a transformation into highly porous aerogels after a solvent exchange with alcohol or other organic solvents followed by SCD.

### 2.3 Controlled Assembly of Nanoscale Building Blocks

**Figure 1:**
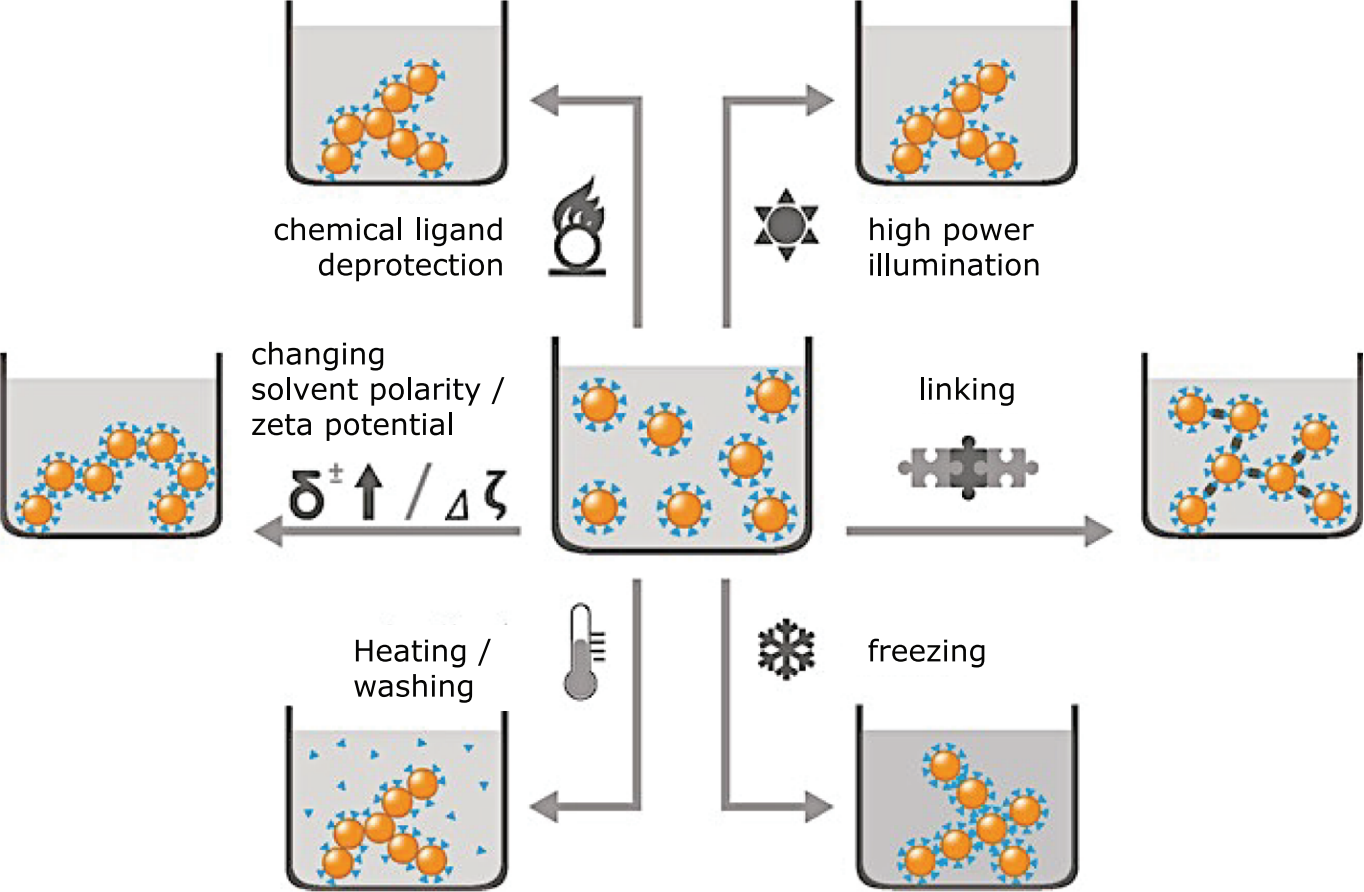
Strategic technique for controlled destabilization of preformed colloidal solutions through various stimuli. Classical gel formation methods use pH, ionic strength, polarity, heating, freezing, or connection via (poly)electrolytes. Modern aerogel manufacturing involves controlled destabilization of preformed sols through chemical ligand deprotection through oxidizing agent addition, high‐power illumination, and washing. Reproduced by permission.^[^ ^1^ ^]^ Copyright 2017, John Wiley & Sons.

In addition to the classical gelation that uses molecular precursors as starting precursors, the aerogel 3D network can also be built through the controlled assembly of colloidal solutions of nanosized building blocks.^[^ ^26^ ^]^ This gelation method was first introduced in 2004^[^ ^27^ ^]^ for developing semiconductor‐based aerogels and then extended to other aerogels.^[^ ^1^ ^]^ **Figure** **1** demonstrates different stimuli that can trigger the gelation process in a colloidal solution. One approach relies on building the gel network through controlled destabilization of highly stable colloidal solution of nanoparticles (NPs) by removing their surface ligands or capping agents by chemical or photochemical oxidation/ligand deprotection approaches. With this approach, the NPs are gradually destabilized and coagulated and build a 3D gel network structure. The freeze‐gelation or ice‐templating process is another physical destabilization approach independent of the surface ligands.^[^ ^28^ ^]^ Here the colloidal solution of highly dispersed particles undergoes solidification by growing the ice crystals inside the colloidal solution to assemble the particles in the area between the ice lamella/crystals, followed by ice crystals defrosting and SCD or in the frozen state through freeze‐drying. In the next step, the self‐supporting assembled aerogel is developed by SCD of the developed gels.

### 2.4 Aging

Aging is the process of obtaining a reinforced silica gel network.^[^ ^2^, ^20^ ^]^ The traditional aging step was a very time‐consuming process based on increasing the number of siloxane bonds between particles. However, the duration of this step was reduced from weeks to days by immersing the base catalyst gel in an ethanol/water mixture at pH 8–9.^[^ ^29^ ^]^ The aging process has been described as a “coarsening” and “Ostwald ripening” mechanism.^[^ ^1^ ^]^ Due to the reversible nature of hydrolysis and condensation reactions, gel network structures tend to become mechanically stable scaffolds by thermodynamically driven rearrangements such as Ostwald ripening. The pore may be roughened due to the phase separation of the structure. Therefore, the average pore size increases by decreasing the crosslinking density of the network structure.^[^ ^30^ ^]^

### 2.5 Drying

Drying, the final step of aerogel fabrication is an essential step in which solvents in the pores of the wet gel are vaporized and removed using different techniques.^[^ ^31^ ^]^ The porosity and surface area of aerogels are known to vary with the drying techniques used.^[^ ^32^ ^]^ In conventional evaporative drying techniques, the capillary pressure generated during this drying method causes the structure to shrink and break.^[^ ^33^ ^]^ For this reason, in the drying step, advanced drying methods such as freeze‐drying,^[^ ^32^ ^]^ carbon dioxide supercritical drying (CO_2_SCD),^[^ ^32^ ^]^ acetone, methanol or ethanol,^[^ ^34^ ^]^ ambient pressure drying (APD),^[^ ^32^ ^]^ and vacuum drying (VD),^[^ ^32^ ^]^ are used to remove solvents from the aerogel without disturbing the pore structure. The comparisons of drying methods are summarized in **Table** **1**.

**Table 1:** The features of some aerogel examples made with different drying methods

Materials	Drying Technique	Volume shrinkage [%]	Density [g cm^−3^]	Thermal conductivity [W mK^−1^]	Pore volume [cm^3^ g^−1^]	Average pore diameter [nm]	BET Surface area [m^2^ g^−1^]	Ultimate Strength [MPa]	Reference
Silica Aerogel/BTESB	APD	26	0.52	0.055	0.81	47.4	155	2.658	[32]
	CO_2_SCD	12	0.29	0.060	3.94	75	209	1.146	
Silica Aerogel/BTMHS	APD	14	0.24	0.075	3.47	261	53	0.786	[32]
	CO_2_SCD	11	0.22	0.052	3.5	170	82	0.523	
Nanocellulose Aerogel	VD	‐	‐	‐	‐	‐	20	‐	[32]
	FD	–	–	–	–	–	66	–	
Carbon Aerogel	APD	–	1.13	–	0.22	–	550	1680	[32]
	CO_2_SCD	–	0.51	–	1.11	–	704	130	
Carbon aerogels	FD	–	–	–	1.341	7.21	843	–	[32]
	VD	–	–	–	1.607	8.53	765	–	
	APD	–	–	–	0.981	7.01	593		
Silica aerogel/carbon microfiber nanocomposites	APD	8.8	0.13	–	–	15.8	775	–	[32]
	CO_2_SCD	5.9	0.1	–	–	10.9	821	–	
Silica aerogel	APD	27.7	0.2	–	–	12.2	864	–	[32]
	CO_2_SCD	9.1	0.125	–	–	10.7	802	–	
Zirconia Aerogel/%8 Yttria	FD	–	–	–	0.066	0.748	483	–	[32]
	CO_2_SCD	–	–	–	3.373	6.638	701	–	
Zirconia Aerogel	FD	–	–	–	0.104	0.593	397	–	[32]
	CO_2_SCD	–	–	–	2.504	9.767	641	–	
Yttria‐stabilized zirconia Gel	APD	–	–	–	–	–	2.8	–	[32]
	CO_2_SCD	–	–	–	–	–	26	–	
Cellulose Nanofiber Aerogels	FD	12.63	0.0363	–	0.856	18–23	106	0.85	[32]
	CO_2_SCD	5.56	0.0201	–	0.720	23–33	296	0.37	
Silica‐based gels	APD	–	0.078	–	–	3.6	426	–	[32]
	CO_2_SCD	–	0.054	–	–	4.4	512	–	

^a)^ APD: Ambient pressure drying; CO_2_SCD: Carbon dioxide Supercritical drying; FD: Freeze Drying; VD: Vacuum drying; BTESB: 1,4‐Bis(triethoxysilyl) benzene; BTMHS: 1,6‐Bis(trimethoxysilyl) hexane.

#### 2.5.1 Supercritical Drying (SCD) Technique

The SCD method is the most frequently applied drying method because it preserves the pores and texture of wet gels. This method removes liquid from the pores of a wet gel in a closed container above the critical temperature and pressure values.^[^ ^35^ ^]^ This drying technique can be performed using either high‐ or low‐temperatures which has been comprehensively reviewed before.^[^ ^2^ ^]^

The SCD method can be the most effective method to prevent mesopore shrinkage and structural collapse during drying.^[^ ^36^ ^]^ Despite these advantages, there is a limit to use on an industrial scale due to the 1) use of high temperature and high pressure, 2) a large amount of solvent, 3) a long processing time, and 4) a high overall cost.

#### 2.5.2 Freeze‐Drying Technique

The freeze‐drying technique has been widely used in the manufacture of aerogels because of its eco‐friendly, economical, and simple process. This technique also provides high porosity with low shrinkage. In this method, the solvent in the wet gel is extracted from the structure by sublimation after freezing at low pressure.^[^ ^37^ ^]^ Compared with SCD aerogels, aerogels prepared by this method have a larger porous structure with greater shrinkage.^[^ ^38^ ^]^ Freeze‐drying is a more widely used method than SCD in aerogel production, specially for biomedical applications, due to its environmental friendliness and easy alignment and control of porous structures.^[^ ^39^ ^]^

#### 2.5.3 Ambient Pressure Drying (APD) Technique

In the APD technique, liquid is removed from a wet gel by evaporating it at ambient pressure at either ambient^[^ ^40^ ^]^ or elevated temperature.^[^ ^41^ ^]^ This is a simple technique for preparing aerogels but results in highly shrunken, less porous materials.^[^ ^42^ ^]^ These formations, which are the most dense and might be cracked, are called xerogels. During the shrinkage of xerogels, newly formed siloxane bonds by the condensation reaction of unreacted hydroxyl/alkoxy groups make the shrinkage irreversible.

Taken together, any drying methods can cause some degree of shrinkage. SCD is the most efficient way to reduce shrinkage in the drying process, but it is the most difficult to commercialize in terms of process cost. In addition, although APD technology is still emerging as the most promising technology overall, it has the disadvantage that the evaporation of atmospheric drying technology may cause harmful air pollution to humans and animals.^[^ ^35^ ^]^ For these reasons, so far, freeze‐drying is considered the most optimal technology in terms of economical, environmental, and production quality (i.e., shrinkage rate).

### 2.6 Post‐Processing: Carbonization

**Figure 2:**
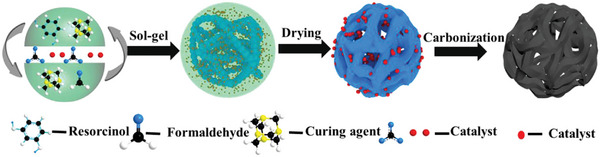
Schematic illustration showing synthesis steps of resorcinol formaldehyde (RF)‐based carbon aerogels. Reproduced with permission.^[^ ^43^ ^]^ Copyright 2018, Elsevier.

After the drying step, pyrolysis/carbonization (e.g., **Figure** **2**) can be used to remove volatile materials and hybridize sp^2^‐carbon atoms. The carbonization step can increase the surface area and, consequently the electrical conductivity of the aerogel where required for a specific biomedical application. However, to further increase the porosity and surface area of the carbon aerogel, physical or chemical activators can be added to the carbon backbone after or during the carbonization step. For example, at high temperatures, physical activators such as steam and CO_2_ can react with carbon to release hydrogen (H_2_), carbon monoxide (CO), and CO_2_, generating structures with high porosity and large pore size.^[^ ^43^ ^]^ Similarly, chemical activators such as alkaline reagents with strong etching ability (e.g., KOH, H_3_PO_4_) can attach to the carbonous precursor during the carbonization step and then be heated at high temperatures to form highly porous structures. An advantage of using chemical activators over physical ones is their lower activation temperature, higher yield, and higher surface area.^[^ ^43^ ^]^

The high‐temperature sintering approach for some ceramic and metal‐based aerogels is relevant during the final materials' post‐treatment densification. Through high‐temperature sintering, binders like organic ligands, particle stabilization agents, and surfactant residues are removed, and the network particles sinter together. Also, for aerogel systems developed by an assembly of 1D nanowires, the high temperature of sintering promoted the chemical bonding between the glass binder and the 1D building block in the gel network.^[^ ^44^ ^]^ This post‐fabrication treatment route is essential for developing ceramic‐based aerogels used as temporary bone implants.^[^ ^45^ ^]^

## 3 Materials

Essentially, the term aerogel defines a distinct geometric structure of matter. It is neither restricted to any material nor to any synthesis process. Hence, the possible variety of materials and, therefore, their diverse applications are almost limitless.^[^ ^1^ ^]^

### 3.1 Inorganic and Organic Materials

Inorganic aerogels are mostly those comprised of tetramethoxysilane (TMOS) or the less toxic tetraethoxysilane (TEOS) through sol‐gel polymerization. This material category is the first type of aerogel in the nineteenth century when Kistler synthesized aerogel for the first time. The aerogels made of these categories of materials are mostly those made of Si‐based monomers and called silica aerogels. There are several detailed review articles dedicated to this family of material for further reading.^[^ ^1^, ^20^ ^]^ In short, these aerogels had been mostly considered for biosensors, pharmaceutical sciences, and diagnostics biotechnology.^[^ ^46^ ^]^

Organic aerogels are mostly those comprised of polymers such as polysaccharides and proteins. For more information, we suggest referring to the review by Al‐Muhtaseb^[^ ^47^ ^]^ about conventional carbon aerogels and the review on nanocarbon aerogel by Ansari.^[^ ^48^ ^]^ These aerogels have been studied for several biomedical applications, such as tissue engineering,^[^ ^49^ ^]^ wound healing,^[^ ^50^ ^]^ regenerative medicine, and drug delivery systems.^[^ ^51^ ^]^

### 3.2 Carbon Materials

Carbon aerogels can also be synthesized from carbon NPs. The synthetic procedure can occur in two ways: 1) direct aerogel formation from carbon‐based NPs precursors, and 2) carbon‐based NP growth on non‐carbonous aerogels. In the following section, for the convenience of understanding, carbon NP‐derived aerogels are divided into CNTs, graphene NPs (GNs), and NDs according to the types of NPs. Each corresponding example and the associated synthesis procedure are described.

#### 3.2.1 Carbon Nanotube (CNT) Aerogels

In 2007, Bryning et al. developed the first aerogel made of single‐walled CNT by dissolving CNT powder (5–13 mg mL^−1^) in water using sodium dodecylbenzene sulfonate (SDBS) as a surfactant.^[^ ^52^ ^]^ The solution was cast into a mold and left overnight to form an elastic gel. The gels were then reinforced with varying polyvinyl alcohol (PVA) concentrations (0.25–1 wt.%) and subsequently dried using freeze‐drying or CO_2_SCD techniques. The CNT aerogel without PVA exhibited a conductivity of 1 S cm^−1^ and a density of 1.4 g cm^−3^. When 1 wt.% PVA was added to the CNT aerogel, electrical conductivity dropped to 10^−5^ S cm^−1^. Although the PVA reinforcement improves the physical properties of CNT aerogels, the decrease in conductivity hinders the electrical application of this aerogel. To tackle this issue, the authors applied 30 s, high‐current (100 mA) pulses to the PVA‐reinforced CNT aerogel and successfully increased the conductivity to 10^−2^ S cm^−1^. This property is desirable for biomedical applications in diagnostic electrical devices that require a low‐density, high‐surface‐area electrical conductor.^[^ ^52^ ^]^

In 2010, Zou et al. prepared ultralight aerogels using multi‐walled CNTs (MWCNTs).^[^ ^53^ ^]^ MWCNTs were dispersed in a mixture of chloroform and poly(3‐hexylthiophene)‐b‐poly(3‐(trimethoxysilyl)propyl methacrylate) (P3HT‐b‐PTMSPMA) to conjugate PTMSPMA on the surface of CNTs. After solvent exchange to methanol, the wet gel was crosslinked with ammonia, and the final solvent was exchanged with water and freeze‐dried to obtain MWCNT aerogels. The aerogels presented a surface area of 580 m^2^ g^−1^ which was around twofold higher than that of original MWCNTs. The obtained aerogel not only had an electrical conductivity of 3.2×10^−2^ S cm^−1^, but was also flexible, porous, and ultralight, making it a promising candidate for pressure and chemical sensing for medical diagnosis.^[^ ^53^ ^]^

In 2016, Shen et al. followed the synthesis protocol of Bryning et al. and were able to prepare low‐cost CNT aerogels with controllable density using SDBS.^[^ ^54^ ^]^ After adjusting the proportion of CNTs, SDBS, and water under ultrasonication, the dispersion turned into a wet gel in 2 days. The wet gels were washed with 1 wt.% PVA at a high temperature (90 °C). After exchanging the solvent with ethanol, supercritical CO_2_ drying (SCD) resulted in the formation of a CNT aerogel. The resulting CNT aerogels exhibited low density in a range of 0.06 to 0.35 g cm^−3^, high elastic modulus of 0.17–0.87 MPa, a surface area of 100 m^2^ g^−1^, and good electric conductivity ranging from 0.56–4.55 S m^−1^ with a pore size of 10–20 nm.^[^ ^54^ ^]^

A study using CNTs to reinforce Ti3C2Tx aerogels through a bidirectional freezing method was reported by Sambyal et al. The hybrid Ti_3_C_2_T_x_/CNT aerogels demonstrated improved robustness, good electromagnetic interference (EMI) shielding, and 9661% improved compressive modulus compared to the original Ti_3_C_2_T_x_ aerogel. The density of the aerogels was 0.016−0.042 g cm^−3^, and the porosity was 93–98%. The high electrical conductivity (9.43 S cm^−1^), along with EMI shielding and high mechanical strength, make these hybrid aerogels an ideal candidate for EMI contamination adsorption.^[^ ^55^ ^]^

In another study, hydrophobic SiO_2_ aerogel was reinforced with CNTs during a one‐pot sol‐gel process. The obtained CNT/SiO_2_ aerogel could withstand a compressive load of 12.6 MPa, which was 90 times greater than the original SiO_2_ aerogel. The resulting aerogel had a pore volume of 2.92 cm^3^ g^−1^ and a density of 0.07 g cm^−3^ and showed a high absorptive capacity to absorb oils 15 times more than its weight. Besides, with a simple heat treatment, the adsorption capacity can be recycled up to 30 times.^[^ ^56^ ^]^

#### 3.2.2 Graphene Aerogels (GAs)

Graphene oxide (GO) is usually a common precursor to generate GAs due to its high dispersibility in an aqueous media. Besides, GO has several hydrophobic and hydrophilic functional groups on the basal plane and edges that can covalently bond with multiple compounds to generate novel materials with various tunable properties.^[^ ^57^ ^]^ GA is considered the least dense or lightest existing solid material with an air content of 99.98%, along with excellent flexibility, electrical conductivity, and a high absorption rate (≈ 850 times more than its weight). These properties make GA a promising material for medical diagnosis applications. The most common synthesis methods of GAs using GO include the hydrothermal reduction process, chemical reduction methodology, and crosslinking.

**Figure 3:**
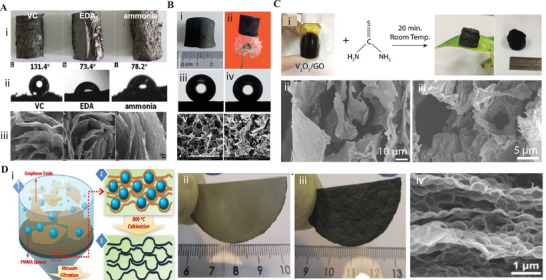
Fabrication methods of GAs. A) i) Photographs of the GA prepared using different reducing agents such as VC, EDA, and ammonia and ii) their corresponding water angels, and iii) SEM images. Reproduced with permission.^[^ ^58^ ^]^ Copyright 2016, Royal Society of Chemistry. B) i,ii) Photographic images of GA. iii,iv) Micrographs of a water droplet on the surface (iii) and cross‐section (iv) of GA. v,vi) Scanning electron microscopy (SEM) images of the surface (v) and cross‐section (vi) of GA. Reproduced with permission.^[^ ^60^ ^]^ Copyright 2015, Royal Society of Chemistry. C) i) Photographic images of vanadium V_2_O_5_/GO hydrogel and the process of converting it to aerogel. ii,iii) SEM images of V_2_O_5_/GO aerogels with different magnifications. Reproduced with permission.^[^ ^61^ ^]^ Copyright 2017, Royal Society of Chemistry. D) i) Schematic illustration demonstrating the fabrication process of MGF. ii,iii) Digital micrographs of GO/PMMA and MGF, respectively. iv) SEM image of MGF. Reproduced with permission.^[^ ^62^ ^]^ Copyright 2012, Royal Society of Chemistry.

Wan et al. fabricated GAs through an eco‐friendly hydrothermal reduction method.^[^ ^58^ ^]^ They studied the effect of various reducing agents, such as ethylenediamine (EDA), ammonia, and vitamin C (VC), on the hydrothermal method of preparing GA. In addition, the change in the properties of GA according to the reaction at various reaction times (4 to 24 h) and at various temperatures (80 to 180 °C) was also analyzed. The reduction reaction removes functional oxygen groups from the GO surface and allows the remaining hydrophobic GNs to self‐assemble into specific sizes and shapes. The GAs reduced by VC exhibited a density of 13.7 mg cm^−3^ and the highest mechanical strength but extremely poor adsorption capacity, whereas GAs reduced by ammonia exhibited a density of 4.9 mg cm^−3^ and the maximum adsorption capacity (160 g g^−1^) but extremely poor mechanical strength (**Figure** **3**A). The GA reduced by ammonia demonstrated a higher surface area (1089 g m^−2^) compared to the GA reduced by the VC (661 g m^−2^). GAs reduced by EDA could maintain good adsorption capacity in a broad hydrothermal reaction window.^[^ ^58^ ^]^ Although the hydrothermal reaction is an easy one‐step process, it requires high pressure, which limits its applicability to mass production.^[^ ^59^ ^]^

Unlike hydrothermal reduction, which requires high temperature and high pressure, the chemical reduction method requires only a reducing agent; therefore, this method can be employed for mass production. Several reducing agents, such as hydrazine, VC, sodium ascorbate, hydrazine, oxalic acid, mercaptoacetic acid, or mercaptoethanol hybridize sp^2^ carbon and promote the self‐assembly of GO.^[^ ^57^ ^]^ Xu et al. fabricated superhydrophobic GA from GO using a chemical reduction method using L‐phenylalanine as a reducing agent. The wet black cylindrical hydrogels were freeze‐dried to develop the GA aerogels similar to what is shown in Figure 3B. The obtained GAs exhibited superhydrophobicity/water repellency, superoleophilicity, very low density (0.05 g cm^−3^, high surface area (117 m^2^ g^−1^), high recyclable absorption capacity (>100 g g^−1^) for both oil and organic solvents, pore size of ≈10 µm, pore volume of 0.991 cm^3^ g^−1^, and excellent mechanical strength (Young's modulus ≈ 66 kPa).^[^ ^60^ ^]^ Although the chemical reduction method is simple, scalable, and eco‐friendly, the resulting GA usually has a small surface area due to the *π*–*π* interactions that occur between graphene layers during processing.^[^ ^57^ ^]^

Crosslinking is another method for GA synthesis commonly used in acidic environments. When the pH of the solution is low, the carboxyl groups of GO are protonated, strengthening the network by hydrogen bonding and forming a stable gel. Crosslinkers such as hydroxyl, nitrogen, oxygen, and oxygen‐containing functional groups can increase hydrogen bonding, electrostatic interactions, and covalent bonding between GO sheets and lead to a stronger microstructure.^[^ ^57^ ^]^ For example, divalent and trivalent metal ions can interact with GO sheets and enhance their self‐assembly and gelation time.^[^ ^57^ ^]^ Yilmaz et al. utilized surface‐functionalized GO precursors containing hydroxy, epoxide, and carboxy groups to synthesize V_2_O_5_/GN aerogels (Figure 3C).^[^ ^61^ ^]^ In the presence of thiourea as a crosslinker, these functional groups enhance the self‐assembly of GO sheets and V_2_O_5_ via in situ sulfurizations during a rapid one‐step sol‐gel process. The resulting V_2_O_5_/GN aerogels had surface area, density, and pore volume of 83.4 m^2^ g^−1^, 0.02 g cm^−3^, and 0.464 cm^3^ g^−1^, respectively. They were suitable for energy storage, and their specific capacitance was 484.0 F g^−1^ at 0.6 A g^−1^. The crosslinker technology is simple, environmentally friendly, and cost‐effective. Still, the reduction reaction and crosslinkers result in less porous GA due to the high stacking behavior between the GN sheets.^[^ ^48^ ^]^

The template‐directed reduction is one of the useful methods for the fabrication of porous GAs because it prevents random interactions and allows control and tuning of the network microstructure. As shown in Figure 3D, Chen et al. prepared a macroporous bubble GN film (MGF) with 91.05% porosity and a pore size of 225 nm using template‐directed reduction. PMMA latex spheres were used as a spacer and hard template. GO hydrosol was mixed with PMMA, followed by vacuum filtration to sandwich PMMA spheres between the GO sheets. After air drying, the resulting grey‐colored PMMA/GO film was removed from the filter and calcinated at 800 °C to eradicate the template and generate a black‐colored freestanding compact GN film. The MGF was flexible and maintained its microstructure after the calcination, and exhibited excellent electrochemical capacities with a high rate (1.0 V s^−1^) and current density of 1.0 A g^−1^.^[^ ^62^ ^]^

GN has also been used as the strongest nanomaterial for the reinforcement of other organic and inorganic polymers.^[^ ^63^ ^]^ Patil et al. made GN‐reinforced SiO_2_ aerogels using molecular dynamics simulations.^[^ ^64^ ^]^ They showed that single‐layer GN had a fourfold higher indentation resistance compared to the original SiO_2_ aerogels. Moreover, the hybrid GN/SiO_2_ nanocomposites exhibited higher stiffness. In addition, the indentation resistance significantly increased as the number of GN layers increased, making the double‐layer GN/SiO_2_ mechanically stronger than a single‐layer GN/SiO_2_ nanocomposite.^[^ ^64^ ^]^ In another study, Zhu et al. fabricated periodic GA microlattices using 3D printing technology(i.e., direct ink writing) and sol‐gel chemistry.^[^ ^65^ ^]^ The composite ink was made of GO solution, hydrophilic silica powder, and RF solution. Silica powder was used as a catalyst, and RF solution controlled the gelation process. The 3D‐printed nanocomposite underwent CO_2_SCD to form GO with RF aerogel. The resulting lightweight aerogels exhibit organized macropores with a pore volume of 2.5 cm^3^ g^−1^, a density of 123 mg cm^−3^, and a large surface area of 704 m^2^ g^−1^ while maintaining mechanical strength, high conductivity (278 S m^−1^), and 90% of capacitance retention.^[^ ^65^ ^]^

#### 3.2.3 Nanodiamond Aerogels (NDAs)

NDA is the least dense diamond, 40 times denser than the air, and has a high specific surface area with a self‐supporting morphology. Owing to its ability to tune the optical index of refraction (1<n<2.4), NDA is the most widely used material for anti‐reflective coatings used on optical device surfaces.^[^ ^66^ ^]^ Pauzauskiea et al. synthesized NDA for the first time from amorphous carbon aerogel precursor in an inert neon environment using a high‐intensity laser in a diamond anvil cell. The high pressure in the anvil cell compressed the aerogel to the range of 21.0 to 25.5 GPa, followed by laser heating to convert the amorphous carbon to a cubic nanocrystalline diamond. The obtained NDA was transparent and could transmit the incident light. Furthermore, it was porous, light‐weighted (density of 0.040 g cm^−3^), and biocompatible.

Manandhar et al. prepared NDA from RF precursors at room temperature and ambient pressure using the rapid sol‐gel method as an alternative method.^[^ ^67^ ^]^ The resulting NDAs were pinkish‐white in color compared to reddish RF aerogel and exhibited a macroporous structure and bulk density of 151 mg cm^−3^ with a pore volume of 0.613 cm^−3^ g^−1^. Due to their large pore size, these NDAs are desirable for energy storage and NASA's Stardust missions, as well as pharmaceutical applications.^[^ ^67^ ^]^

Similar to the other carbon‐based NPs, NDA has also been used for the reinforcement of other aerogels. For example, Mitura et al. fabricated SiO_2_ aerogels coated with a diamond‐like carbon layer using a radiofrequency plasma chemical vapor deposition method.^[^ ^68^ ^]^ The coated aerogel is semi‐transparent, impervious to alkaline and acidic environment, and has the potential for use in electronics and sensor applications.^[^ ^68^ ^]^ Roldán et al. prepared GA/ND hybrids from GO precursors using the self‐assembly hydrothermal reduction method.^[^ ^69^ ^]^ Incorporating ND into the network prevents the GN sheets from restacking while at the same time avoiding the aggregation of ND. The obtained GA/ND hybrids with low ND content (2 wt.%) demonstrated 18% higher activity than the original GAs. Because GA/ND hybrids are self‐standing and highly porous morphology, they are desirable as catalysts or adsorbents in flow systems.^[^ ^69^ ^]^ Examples and characteristics of carbonous aerogels are summarized in **Table** **2**.

**Table 2:** Summary of carbonous aerogels’ characteristics

Material	Drying Approach	Pore Size [nm]	Pore volume [cm^3^ g^−1^]	Density [g cm^−3^]	Surface Area [m^2^ g^−1^]	Application	Ref.
RF carbon aerogels	Ambient pressure drying at 50 °C followed by carbonization at 1000 °C.	13.3	3.41		2057	Capacitive deionization and electrosorption of copper ions	[70]
RF/Al_2_O_3_	Ambient pressure 80 °C drying			0.077–0.112	453.26–722.75	Electrochemical applications	[71]
CNT/PVA	Supercritical CO_2_ fluid drying	10–20		0.06–0.35	100	Electrical devices	[54]
CNT/Ti_3_C_2_T_x_	Freeze‐drying			0.016−0.042		Adsorption of EMI pollution	[55]
CNT/SiO_2_	Dried at 150 °C		2.92	0.07		Oil removal	[56]
GA	Freeze‐drying	≈10 µm	0.991	0.05	117	Oil‐absorption and oil‐water separation	[60]
V_2_O_5_/GN	Freeze‐drying		0.464	0.02	83.4	Electrochemical	[61]
MGF	Freeze‐drying	225				Electrochemical application	[72]
GO‐RF	CO_2_SCD		2.5	123	704	Catalysis, desalination, and other filtration/separation applications	[65]
NDA	CO_2_SCD	<60		151	589 ^a)^Related to microporous	Pharmaceutical applications, energy storage, NASA's Stardust missions	[67]

### 3.3 Hybrid Materials (Organic/Inorganic)

Hybrid materials can be classified into two main categories, denoted as “class I hybrids” and “class II hybrids”, based on their interactions with organic and inorganic compounds. The organic and inorganic moieties of class I hybrid materials are connected through weak physical interactions like electrostatic, van der Waals, and hydrogen bonds. On the other hand, in class II hybrids, the two moieties are connected via strong chemical interactions such as covalent bonds or ionic‐covalent bonds.^[^ ^73^ ^]^ In other words, class I hybrids are simply mixing organic and inorganic compounds under a sol‐gel system. In contrast, class II hybrids require the functional groups of organic components to be conjugated with inorganic precursors. Therefore, class II hybrids exhibit a more precise microstructure and improved physicochemical stability compared to class I hybrids due to the chemical conjugation.^[^ ^4^ ^]^

In 1994, Novak and coworkers fabricated the first low‐density hybrid class I and II aerogels by incorporating organic polymers into the silica (SiO_2_) aerogels.^[^ ^74^ ^]^ Among the several linear polymers that were physically added to the SiO_2_ gel, poly(2‐vinylpyridine) (PVP) showed the most stable structure with minimal leaching from the SiO_2_ network.^[^ ^4^ ^]^ Other polymers such as poly(methyl vinyl ether),^[^ ^75^ ^]^ poly(viny1alcohol) (PVA),^[^ ^76^ ^]^ poly(N‐vinylpyrrolidone),^[^ ^77^ ^]^ poly(ethylene oxide) (PEO),^[^ ^78^ ^]^ poly(methyl methacrylate) (PMMA),^[^ ^79^ ^]^ cellulose,^[^ ^80^ ^]^ and Xanthan^[^ ^5^ ^]^ could not integrate well into the SiO_2_ framework after physical mixing and were leached out during the solvent evaporation and drying procedures.^[^ ^4^ ^]^ To address this problem, researchers have used the co‐condensation reaction of PMMA‐co‐(3‐(trimethoxysilyl)propyl methacrylate) (PMMA‐TMSPM) or silanol‐terminated polydimethylsiloxane (PDMS)^[^ ^81^ ^]^ to covalently crosslink organic and inorganic polymers.^[^ ^4^ ^]^ The synthesized hybrid aerogels showed low hydrophilicity, improved optical density, and high toughness and compression strength.^[^ ^74^ ^]^ Similar approaches have been employed to improve the quality of fragile pristine SiO_2_ aerogels using different crosslinkers. Leventis et al. reported the crosslinking of SiO_2_ hydrogels with poly(hexamethylene diisocyanate) using a post‐gelation doping method. As a result, it was possible to form a lightweight SiO_2_ aerogel monolith that did not collapse in contact with liquid. The aerogel made of SiO_2_‐conjugated diisocyanate (di‐ISO) was translucent with tunable properties depending on the concentration of di‐ISO and the density of the aerogel. The SiO_2_ monoliths with a density of 0.447 g cm^−3^ could undergo a 100‐fold higher load (≈15 kg) before break‐failure compared to the pristine SiO_2_ aerogel (failure load of ≈ 120 g).^[^ ^82^ ^]^

The above‐mentioned chemical hybridization significantly enhanced the mechanical properties of SiO_2_ aerogel. However, in this process, organic polymers were introduced into the solid network only after the gelation process. Such a post‐gelation hybridization approach reduces the homogeneity of the hybrid aerogels due to the slow diffusion that drastically increases the reaction time.^[^ ^83^ ^]^ To address this issue, Duan et al. reinforced SiO_2_ aerogels by hybridizing polyurethane (PU) into the network before gelation using 3‐Aminopropyltriethoxysilane (APTES) as a crosslinking agent.^[^ ^84^ ^]^ The technique significantly reduced the processing time and improved compression strength by a factor of five while reducing SiO_2_ aerogel density by 60%. Similarly, Talebi Mazraeh‐shahi et al. synthesized uniform PU/SiO_2_ hybrid aerogels using a one‐step sol‐gel method. The authors observed a PU concentration‐dependent effect on the porosity and physicochemical properties of the aerogel. The addition of a high concentration of PU aqueous dispersion (3 v/v%) to the SiO_2_ solution resulted in a low porous structure (61%) with a smaller surface area (535 m^2^ g^−1^).^[^ ^85^ ^]^

**Figure 4:**
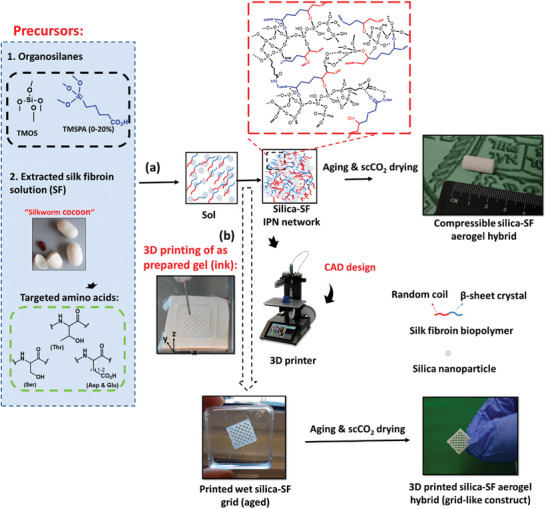
a) Schematic illustration exhibits a one‐pot in situ sol‐gel‐ self‐assembly fabrication method of SiO_2_/SF aerogels from SF solution and organosilanes (i.e., TMSPA and TMOS) using CO_2_SCD (scCO_2_). b) 3D printing of SiO_2_/SF aerogel used as an ink followed by post‐printed processing to fabricate a hybrid grid‐like construct. Reproduced with permission.^[^ ^92^ ^]^ Copyright 2018, American Chemical Society.

SiO_2_ aerogels can be hybridized with biopolymers as well as synthetic polymers. Biopolymers such as proteins, carbohydrates, or plant oils are of interest because of their accessibility, low cost, and biocompatibility.^[^ ^4^, ^86^ ^]^ In addition, various biopolymers such as pectin,^[^ ^5^, ^86^ ^]^ chitosan (CS),^[^ ^87^ ^]^ lignin,^[^ ^88^ ^]^ silk,^[^ ^18^ ^]^ cellulose,^[^ ^89^ ^]^ and alginate^[^ ^90^ ^]^ have been successfully used in the synthesis of hybrid aerogels.^[^ ^91^ ^]^ As an example of applying pectin to the aerogel, a one‐pot sol‐gel method was used to synthesize an adiabatic SiO_2_‐pectin hybrid aerogel by controlling the pH value of two phases; i) aqueous solution of water‐glass‐derived silicic acid and ii) pectin with high methoxy content.^[^ ^86^ ^]^ The hybrid aerogels produced at pH 1.5 with 5 wt.% pectin had reduced dust release, loss of hydrophobicity (θ < 90°), improved mechanical properties (compressive modulus of ≈ 2.5 MPa), and reduced thermal conductivity (14.2 mW m^−1^K^−1^) compared to pristine SiO_2_ aerogel. In another study, silica‐CS aerogel was produced through a sol‐gel reaction with hexamethyldisiloxane and CS from agricultural waste.^[^ ^87^ ^]^ The aerogels fabricated using the APD technique exhibited a bulk density of 0.441 g cm^−3^, a pore surface area of 237.4 m^2^ g^−1^, and a pore volume of 0.286 cm^3^ g^−1^. Maleki et al. reported the fabrication of hybrid silica/silk fibroin (SiO_2_/SF) aerogels using the one‐pot acid‐catalyzed sol‐gel method.^[^ ^92^ ^]^ The SiO_2_/SF hybrid aerogel was fabricated using a phase separating inhibitor (i.e., cetyltrimethylammonium bromide; CTAB) reaction, solvent exchange, and SCD process (**Figure** **4**). The resulting hybrid aerogels showed improved material properties such as ≈ 90% porosity, a pore diameter of 37–79 nm, an increased surface area of ≈400–800 m^2^ g^−1^, a density of ≈0.11–0.2 g cm^−3^, an increased compressive modulus of threefold, and thermal conductivity of 0.033–0.039 W m^−1^K^−1^. The tunable mechanical and physical properties and printability are promising characteristics for multifunctional SiO_2_/SF aerogel fabrication.^[^ ^92^ ^]^

Cellulose, especially in the form of nanofiber, has been used with inorganic materials such as SiO_2_ precursors to support the inorganic networks and increase the aerogels' mechanical properties.^[^ ^4^ ^]^ For instance, an ultralight electrospun silica‐cellulose diacetate (CDA)‐based hybrid nanofiber aerogel was fabricated using a sol‐gel process.^[^ ^80^ ^]^ The silica precursor (TEOS) solution was slowly mixed with the CDA solution at various ratios and stirred for 1 h at 70 °C. The mixture underwent sol‐gel processing while electrospinning at a flow rate of 0.5 m h^−1^ and voltage of 20 kV using a 22‐G spinneret. The electrospinning process allows the homogenous distribution of silica and CDA and creates mechanically robust 3D nanofibers. The resultant nanofibers were chopped under high‐speed homogenization and froze cast in a nonsolvent followed by freeze‐drying to form nanofiber aerogels. At the final step, thermal crosslinking of the nanofiber aerogels yielded mechanically and thermally stable hydrophobic aerogels demonstrating low density (10 mg cm^−3^), high porosity (≈98%), and high affinity to oil.^[^ ^80^ ^]^

In another approach, mesoporous SiO_2_‐lignin hybrid aerogel was fabricated by the sol‐gel method in situ using lignin extracted from rice husks.^[^ ^93^ ^]^ This study investigated the effects of various synthetic conditions, such as lignin to SiO_2_ ratio, reaction temperature, reaction time, and pH conditions, on the pore structure of hybrid aerogels. The lignin/silica ratio of 2:3, the temperature of 90 °C, pH ≈3, and the reaction time of 6 h were found to be optimum conditions for the fabrication of hybrid aerogels. In optimum conditions, the resulting silica/lignin aerogels exhibited a spherical pore shape with a 60 nm diameter and 471.7 m^2^g^−1^ of specific surface area.

**Figure 5:**
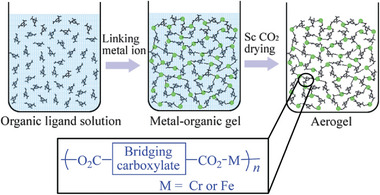
Schematic illustration demonstrating the synthesis of sponge‐like porous metal‐organic hybrid aerogel. Reproduced with permission.^[^ ^94^ ^]^ Copyright 2012, Royal Society of Chemistry.

In addition to SiO_2_ hybrid aerogels, metal‐organic hybrid aerogels have become attractive due to their potential application in adsorption, chemical sensors, and separation technologies.^[^ ^94^ ^]^ For example, Xiang et al. prepared highly porous sponge‐like hybrid aerogels by mixing Cr^3+^/Fe^3+^ salt solution and carboxylic acid at high temperatures (≈80 °C) followed by CO_2_SCD (**Figure** **5**).^[^ ^94^ ^]^ The gel formation rate was tightly dependent on the type of solvent, reaction temperature, metal‐to‐organic material ratio, and precursor concentration. Such metal‐organic aerogels exhibited micro‐ and mesoporosity, which could be tuned according to the concentration of the reactants. Depending on the reactants concentration, the surface area, meso/micropore size, and pore volume changed in the range of 484 to 1090 m^2^ g^−1^, 3.23/1.68 to 33.3/1.88 nm, and 0.23–1.36 cm^3^ g^−1^, respectively.^[^ ^94^ ^]^ Additionally, examples and properties of hybrid aerogels are summarized in **Table** **3**.

**Table 3:** Summary of characteristics of hybrid aerogels

Material	Sol‐gel Approach	Drying Approach	Porosity [%]	Pore Size [nm]	Pore Volume [cm^3^ g^−1^]	Density [g cm^−3^]	Surface Area [m^2^ g^−1^]	Application	Ref
Pectin (5 wt.%) ‐Silica	One‐pot sol‐gel pH:1.5	CO_2_SCD	92	29	5.9	0.13±0.01	827	Thermal insulator	Zhao et al.^[^ ^86^ ^]^
Chitosan ‐Silica hybrid aerogel	Sol‐gel process at ambient pressure	Ambient pressure 80 °C drying	–	–	0.286	0.441	237.4	=	Ebisike et al.^[^ ^87^ ^]^
Silica ‐Silk fibroin	One‐step sequential sol‐gel processes of acid‐catalyzed	CO_2_SCD	92–99%	37–79	≈4.5–8	0.11 – 0.2	400–800	Thermal insulator or as an open‐cell for regenerative medicine application	Maleki, et al.^[^ ^92^ ^]^
Alginate ‐Silica	Conventional two‐step sol‐gel	CO_2_SCD	–	8–16.5	1.1–4.1	–	≈ 407–905	Drug delivery	Ulker et al.^[^ ^95^ ^]^
Silica‐Cellulose diacetate nanofiber	Sol‐gel electrospinning process	Freeze dry	>98%	Primary pores of 4–5 µm, secondary pores of 40–50 µm	–	≈ 10	–	Oil spill cleaning, Thermal insulator	Pirzada et al.^[^ ^80^ ^]^
Silica ‐lignin	One‐step sol‐gel process	Dried at 100 °C	–	60	1.8–3.25	–	471.4	Environmental decontamination	Qu et al.^[^ ^93^ ^]^

## 4 Additive Manufacturing of Aerogels

The use of AM^[^ ^96^ ^]^ for the fabrication of porous scaffolds in the form of aerogels has been reviewed by Titek et al. in 2021.^[^ ^97^ ^]^ According to Titek et al., incorporating AM technologies (microfluidics, micro‐extrusion‐based printing, stereolithography (SLA) with digital light processing (DLP), and droplet‐jetting printing) for the fabrication of aerogels provides some advantages such as the capability of optimizing the properties (physical and chemical) of the fabricated aerogel structures.

As discussed by Tetik et al., for example, in tissue engineering applications, the scaffold should possess interconnecting pores to favor tissue integration and vascularization, having an appropriate surface chemistry for cell attachment, differentiation, and proliferation. The use of 3D printing for aerogel fabrication followed by drying (such as FD or SCD) provides hierarchical porous aerogel‐based scaffolds with improved cellular behavior. In this section, the use of AM to fabricate aerogels for a wider biomedical application (including tissue scaffold) has been reviewed.

### 4.1 Microfluidics

Microfluidic systems can manipulate and process small volumes of fluids and reagents through microchannels. Microfluidic systems have a variety of uses in biomedical applications, including cell separation,^[^ ^98^ ^]^ droplet generation,^[^ ^99^ ^]^ particle synthesis,^[^ ^100^ ^]^ lab‐on‐a‐chip,^[^ ^101^ ^]^ and chemical industries.^[^ ^102^ ^]^ Among the applications mentioned above, it has emerged as a powerful tool for uniform droplet generation for particle synthesis. By controlling fluid flows in microfluidics, monodisperse microdroplets can be generated to make homogenous particles.^[^ ^99^ ^]^

Microfluidic channels are typically fabricated using lithography methods and are made of elastomeric polymers such as PDMS.^[^ ^103^ ^]^ Droplet generation in microfluidic systems offers promising applications in particle generation, drug delivery, and cell encapsulation.^[^ ^104^ ^]^ Each droplet serves as a micro‐reactor with a high surface‐to‐volume ratio, providing fast and efficient reaction and diffusion of materials through the droplet.

The emulsion method can be performed via droplet‐based microfluidic technology for the particle generations, including microgels with narrow size distribution and high reproducibility.^[^ ^105^ ^]^ Therefore, one approach to better control the size and size distribution of the particles is to utilize microfluidic devices for droplet generation. The ability to control channel size, flow, and shear conditions allows the generation of monodisperse particles of different sizes for various medical applications. While micro‐emulsion methods generate particles from bulk solutions with a wide size distribution, microfluidic systems provide independent control of each droplet, efficiently producing large volumes of monodisperse particles while using only a small amount of material.^[^ ^106^ ^]^

**Figure 6:**
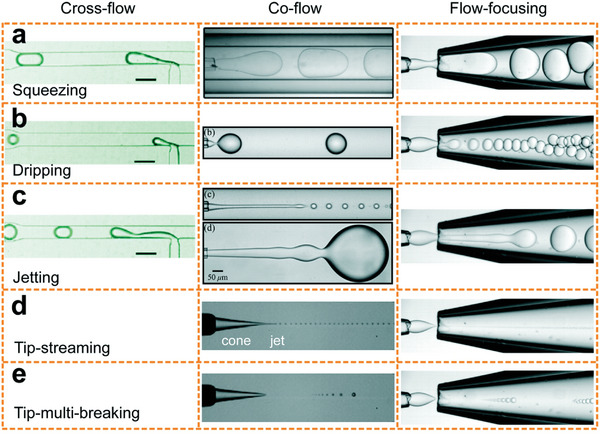
Droplet generation methods and different regimes for generation droplets in crossflow, co‐flow, and flow‐focusing methods. Different regimes for droplet generation. Reproduced with permission.^[^ ^106^ ^]^ Copyright 2017, American Chemical Society.

Most microfluidic devices use viscous shear forces to generate droplets. They are fabricated with a variety of geometries for channels, including cross‐flow (T‐junction),^[^ ^107^ ^]^ co‐flow,^[^ ^106^ ^]^ and flow‐focusing^[^ ^108^ ^]^ (**Figure** **6**) to attain the required shear force fields.^[^ ^99^, ^102^ ^]^ The continuous and dispersed phase flow has parallel streams in the co‐flow configuration. The 2D co‐flow configuration can be fabricated via soft lithography,^[^ ^109^ ^]^ whereas 3D co‐flow geometry for channels is usually created via spatial configurations of the glass capillaries.^[^ ^110^ ^]^ Microfluidic‐based approaches for droplet generation can be classified into two groups: passive approaches and active approaches. Passive methods for droplet generation depend on the geometry of the channel. As examples, three standard passive methods of generating droplets are widely used, including cross‐flow (T‐junction), co‐flow, and flow‐focusing methods (Figure 6). On the other hand, active droplet generation methods use external forces, including magnetic and electric forces as well as mechanical and thermocapillary‐based methods to generate droplets.^[^ ^111^ ^]^ Passive droplet generation methods can be classified into five regimes: squeezing, jetting, dripping, tip‐streaming, and tip‐multi‐breaking (Figure 6). The droplet generation mechanism is based on squeezing, dripping, and jetting regimes (Figure 6) and can be controlled using different geometries, including co‐flow, cross‐flow (T‐junction and Y‐junction), and flow‐focusing configurations.^[^ ^112^ ^]^ Details of the microfluidic droplet generation methods and the regimes of droplet generation can be found in the review paper by Zhu et al.^[^ ^106^ ^]^

Aerogel microparticles are attracting attention because they are simple to produce and are easy to handle, and avoid inflammatory reactions.^[^ ^46^ ^]^ These microparticles with a particular size (50−200 µm) could be beneficial for applications such as ion adsorption or transport, removal of hazardous liquids, as well as drug delivery. Especially in the drug delivery aspect, the short diffusion path and large surface area of aerogel microparticles can be advantageous.^[^ ^100^ ^]^ Aerogel microparticles originated from numerous bio‐based polysaccharide materials, including CS,^[^ ^113^ ^]^ chitin,^[^ ^114^ ^]^ alginate,^[^ ^115^ ^]^ cellulose,^[^ ^116^ ^]^ agar,^[^ ^117^ ^]^ and starch^[^ ^118^ ^]^ have been produced.^[^ ^119^ ^]^

The production of aerogels through droplet‐based microfluidics requires the generation of hydrogels in microfluidic systems, followed by the drying process while maintaining the porous structure of particles. In general, aerogel microparticles are produced through an emulsion polymerization of precursor sol droplets and the use of surfactants. They are stabilized in a series of immiscible phases. The sol droplet is fabricated from a Rayleigh instability state created by stream breakup created by mechanically mixing the two‐phase stream.^[^ ^100^ ^]^ Due to the different shear rate distributions in mechanical mixing and coalescence between particles, the resulting particle size distribution can be broad. Another challenge in making these microparticles is achieving suitable stability for emulsion, especially for systems that require more time for gelation. The conventional droplet‐based microfluidic systems for the production of hydrogels are based on water‐in‐oil (W/O), oil‐in‐water (O/W), or oil‐in‐oil (O/O) emulsions using surfactants as stabilizers.^[^ ^120^ ^]^ For aerogel microparticle production using W/O and O/W emulsion methods, 1) moisture‐sensitive monomeric polymers such as polyimide, or 2) polymers with significant solubility between oil and water are not suitable. In addition, O/O emulsions can result in unstable aerogel microparticles if the surfactants are poorly bound or if the polymer used exhibits significant mutual solubility in the oil phase.^[^ ^121^ ^]^ The similarity of these systems lies in the fact that particles are generated in microfluidic channels, and the sol‐gel transition process occurs outside the microfluidic system.

**Figure 7:**
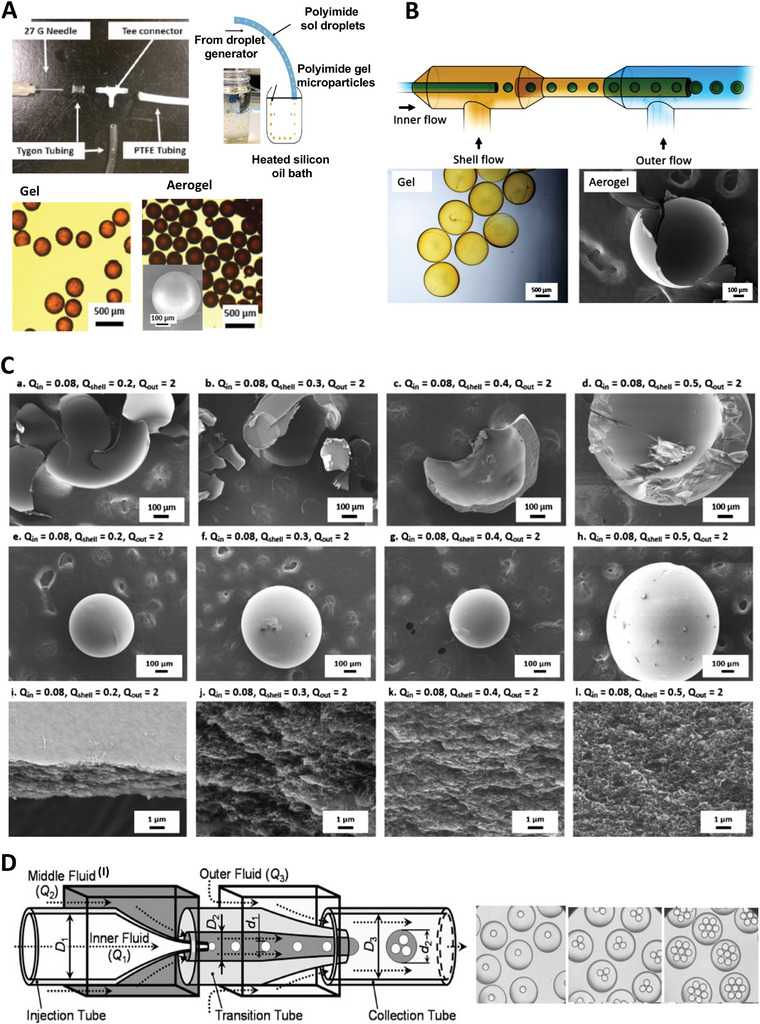
A) Droplet generator system and schematic of gelation in the silicone oil bath. Generated spherical hydrogel and aerogel microparticles are presented below. Reproduced with permission.^[^ ^100^ ^]^ Copyright 2019, American Chemical Society. B) Schematic of a droplet‐based microfluidic system for the generation of core‐shell aerogels. Optical and SEM images of gels and fractured core‐shell aerogel microparticles are presented. Reproduced with permission.^[^ ^123^ ^]^ Copyright 2020, Elsevier. C) Representative SEM images of core‐shell particles to display the effects of Q_shell_ on shell thickness. Flow conditions are shown above each SEM image. Images in (a–d) indicate the shells of intentionally broken core‐shell particles at 100 magnification, (e–h) display the entire core‐shell particles at 65 magnification, while (i–l) display the shell walls of the respective particles at 10 000 magnification, highlighting the porous structure of the shell. Reproduced with permission.^[^ ^123^ ^]^ Copyright 2019, Elsevier. D) Triple emulsion microfluidic design with the optical micrographs of double emulsion droplets containing different numbers of single emulsions. Left image: Reproduced with permission.^[^ ^120^ ^]^ Copyright 2008, Elsevier. Right image: Reproduced with permission.^[^ ^125^ ^]^ Copyright 2007, Elsevier.

Teo et al. developed a simple O/O microfluidic system without using a surfactant to fabricate polyimide aerogel microparticles in the range of 200–1000 µm.^[^ ^100^ ^]^ This work was one of the first to use a microfluidic system to generate aerogel microparticles utilizing polyimide (**Figure** **7**A). The polyimide sol produced in Dimethylformamide (DMF) was converted to droplets suspended in silicone oil. To accelerate the sol‐gel conversion and imidization reaction, the droplets were directed into a hot silicone oil bath at 80 °C for a rapid sol‐gel transition process to generate spherical, individual gel microparticles without coalescence. The microparticles were isolated and dried using the CO_2_SCD technique at 50 °C to attain aerogel microparticles with pore volume, porosity, and surface area of 18.2 ± 1.5 cm^3^ g^−1^, 96.0 ± 0.3%, and 369 m^2^ g^−1^, respectively. The flow rates of the dispersed and continuous phases had a significant effect on the microparticle size distribution. Through this study, the authors tried to solve the problems of aerogel microparticle synthesis, including the large size of the particles, wide size distribution, and low stability of O/O emulsions. They also suggested a surfactant‐free method for particle formation, which eliminates the need for surfactant removal through additional washing.^[^ ^122^ ^]^

In another work, Teo et al. used a microfluidic platform to fabricate core‐shell polyimide aerogel microparticles.^[^ ^123^ ^]^ They generated these particles in both surfactant‐free and surfactant‐based approaches through an O/O/O emulsion setup. In their work, by assembling two co‐flows in a microfluidic device, an emulsion is produced by sequential and stepwise emulsifying a co‐flowing core and organic shell liquid (Figure 7B). The polyimide sol entered the channel as the shell liquid underwent enhanced polymerization in a heated silicone oil bath, producing a porous polyimide shell around the silicone oil core, preventing droplet rupture or coalescence. After isolating the core‐shell gel particles with SCD, core‐shell aerogel microparticles were obtained. The shell thickness and diameter of hollow microparticles were studied by varying the flow rates of liquid and the viscosity of the shell liquid (Figure 7C).^[^ ^99^, ^123^ ^]^ They observed that increasing the shell flow rate (Q_shell_) did not significantly affect the average diameter of gel microparticles; however, the size distribution widened significantly. Moreover, the increase in Q_shell_ leads to a larger shell thickness. The increase in shell thickness is evident in the SEM images shown in Figure 7C(a–d). SEM images showing the whole core‐shell aerogel microparticles are shown in Figure 7C (e–h), and the porous structure of the polyimide shell can be seen in Figure 7C (i–l). They showed that the thickness of the shell and the diameter of particles were highly dependent on the shear rate and the ratio of the inner‐to‐shell flow rates. Additionally, the generated particles showed a broad size distribution, and the addition of surfactant to the polyimide sol delayed the bake‐off of the droplets while increasing the size of microparticles and decreasing the mesoporous volume. In another surfactant‐free approach, Yao et al. proposed the production of multi‐hollow polyimide gel and aerogel microparticles from O/O emulsions in a surfactant‐free scheme under a dripping regime within a microfluidic device. These particles offer great utility in thermal insulation materials. Droplets are generated without clogging the channel in microfluidic devices using tip‐streaming and jetting modes of droplet flows. In addition, they studied a method for producing polyimide gel particles by controlling the flow rates of the dispersed and continuous phases using a microfluidic device. The polyimide aerogel microparticles obtained after SCD exhibited mesoporous structures with high surface area, making them excellent for thermal insulation applications.^[^ ^124^ ^]^

In another research, Chu et al. produced thermoresponsive hydrogel microcapsules via the W/O/W/O triple emulsion technique (Figure 7D).^[^ ^120^, ^125^ ^]^ Their microfluidic channel contains three transition tubes—the inner aqueous fluid forms droplets in the middle oil phase fluid containing the reaction accelerator. Then, the outer aqueous fluid containing monomer, initiator, and crosslinker enters the tube, making a shell of reaction accelerator around the water droplets. Subsequently, the polymerization is initiated by the diffusion of monomer into the outer shell, and a solid capsule is generated. The generated microcapsules contain a hydrogel shell encapsulating the oil core with water droplets. Because the inner core provides controlled thermosensitive release for encapsulated biomolecules, it can be loaded with drug molecules for drug delivery purposes. Water droplets in the core tend to evaporate from the particles by increasing the temperature from 25 to 50 °C, causing the particles to shrink. However, the inner oil is incompressible, so the shell of the hydrogel breaks, releasing water droplets and drug molecules.

**Figure 8:**
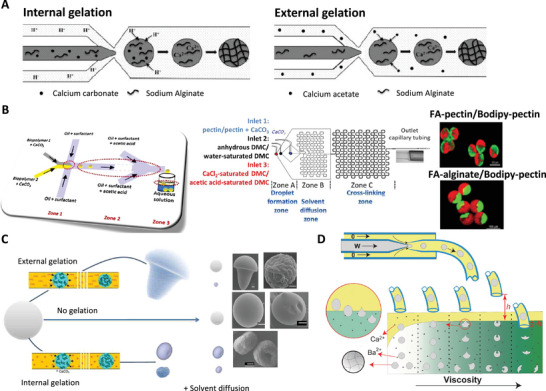
A) Formation of alginate microgels in microfluidic systems based on internal and external relations. Reproduced with permission.^[^ ^6^ ^]^ Copyright 2017, Taylor & Francis Online. B) Schematic of the microfluidic system with flow‐focusing configuration for generation of hemisphere Janus pectin hydrogel microparticles, Confocal images of fluorescein‐amine labeled (FA)‐alginate/Bodipy‐pectin hetero Janus and FA‐pectin/Bodipy‐pectin homo Janus microparticles. Reproduced with permission.^[^ ^129^ ^]^ Copyright 2015, Elsevier. C) Tuning gelation process to produce pectin and alginate with different morphologies. Reproduced with permission.^[^ ^130^ ^]^ Copyright 2014, American Chemical Society. D) Tuning the gelation process to produce microgels with different morphologies. Reproduced with permission.^[^ ^128^ ^]^ Copyright 2012, AIP Publishing.

The sol‐gel transition can also be performed inside the microfluidic channels through internal or external gelation (**Figure** **8**A) by adding a crosslinking agent. For example, both approaches can be utilized, especially for alginate gelation.^[^ ^6^ ^]^ The sodium alginate droplets contain calcium carbonate (CaCO_3_) during internal gelation, and the continuous phase entering from the side channel contains acetic acid. The diffusion of acetic acid into the particles causes the particles to gel.^[^ ^126^ ^]^ However, in external gelation, the continuous oil phase contains calcium acetate, which diffuses into the aqueous droplets of sodium alginate. The release of Ca^2+^ ions initiates gelation in the droplets.^[^ ^127^ ^]^

Microfluidic systems can also be utilized to generate non‐spherical hydrogels of various sizes, scales, and shapes (disc‐shaped, mushroom‐shaped, and erythroid‐shaped), which can be used for a variety of medical applications.^[^ ^128^ ^]^ Marquis et al. developed a flow‐focusing microfluidic chip to generate hemisphere polysaccharide hydrogel microparticles containing two pectin‐pectin hemispheres (homo Janus) or two pectin‐alginate hemispheres (hetero Janus).^[^ ^129^ ^]^ Their microfluidic chip consists of three different zones for droplet formation, solvent diffusion, and crosslinking (Figure 8B). An aqueous solution of two biopolymers (pectin or alginate) mixed with CaCO_3_ used as a crosslinker was contacted with the continuous oil phase at the flow‐focusing junction to form W/O emulsion droplets in the oil. Dimethyl carbonate (DMC) was utilized as the continuous phase with zero saturated water content. Acetic acid was added to the continuous oil flow and diffused into the droplets in the second zone. Finally, as the acetic acid diffuses into the droplets and the pH decreases, calcium ions are released, and the calcium crosslinks in the droplets. This also resulted in crosslinking of polysaccharide chains and internal gelation of biopolymers in the third region. The external diffusion of calcium ions also achieved the gelation process into the droplets by adding aqueous calcium chloride (CaCl_2_) solution to the DMC continuous phase. The generated microparticles were collected in an aqueous CaCl_2_ bath. The pectin‐alginate and pectin‐pectin hydrogel microparticles were characterized by fluorescence labeling. The aerogels were finally obtained by heating the hydrogel particles above the critical point of CO_2_ at 20 °C and 50 bar. In another study, they demonstrated that the structure and shape of the final product could be controlled by applying different crosslinking methods (i.e., internal CaCO_3_ or external CaCl_2_). In addition, the degree of shrinkage of the aerogel was affected by the moisture content of the continuous DMC phase.^[^ ^130^ ^]^ These steps allowed them to finally create aerogel microparticles with spherical, mushroom‐shaped, oval, and donut‐shaped structures (Figure 8C).

Hu et al. fabricated alginate microgels in various shapes, such as hemispherical, red blood cell‐like, and mushroom‐shaped, using a microfluidic system of an external ion crosslinking method.^[^ ^128^ ^]^ They first prepared water droplets containing sodium alginate in a continuous oil phase of n‐decanol with Span‐80 as a surfactant. The droplets were then collected in a biphasic oil/water bath for gelation. The upper and lower phases contained n‐decanol with Span‐80 and barium diacetate or calcium diacetate, respectively, as crosslinkers. The addition of glycerol controlled the viscosity of the bottom phase. The formation of spherical or hemispherical microgels was controlled by adding CaCl_2_ to the upper phase as a pre‐crosslinking agent. They showed that various morphologies of microgels could be generated by adjusting the gelation condition, including the crosslinkers, bath viscosity, interfacial tension, and collection height (Figure 8D). They prepared aerogels by freeze‐drying, which did not change the morphology of the particles. The drug release from drug‐loaded particles was observed depending on the morphology of the aerogel.

Silica‐based aerogels with 80–99.8% porosity have pore sizes of 10–70 nm.^[^ ^98^ ^]^ Silica‐based aerogel can be applied for thermal separation due to its low thermal conductivity of about 0.02 W m^−1^ K^−1[^ ^98^ ^]^ and its large inner surface area (> 2000 m^2^ g^−1^).^[^ ^131^ ^]^ These aerogels can become superhydrophobic when the surface is modified with hexamethyldisilazane (HMDS).^[^ ^98^ ^]^ Consequently, incorporating silica aerogels into microfluidic devices can be attractive in various MEMS applications such as surface modifications, catalysis reactions, and optical measurements. It can also be useful as a boundary between liquid and gas for cell culture in lab‐on‐a‐chip systems. Silica‐based aerogels tend to shrink dramatically during aging and drying processes, at 14% and 51%, respectively.^[^ ^98^ ^]^ Such shrinkage can cause breakage or cracking of the gel and prevent cavity filling of the MEMS device. Reede et al. reported a technique to incorporate silica aerogels into microchips as monoliths.^[^ ^132^ ^]^ They used the sol‐gel method to produce a gel from tetraethyl orthosilicate. Polyethylene glycol and long aging times were exploited to reinforce the matrix and minimize gel shrinkage. This method provided a strong alcogel structure with high stiffness that resists high pressure through a subcritical drying process. The resulting aerogels had a porosity of 85% with a pore size of about 50 nm and a contact angle of 136°.^[^ ^132^ ^]^

In summary, microfluidic systems can provide good control of particle size and produce hydrogels with various shapes and morphologies using different gelation mechanisms outside or inside microfluidic channels. In addition, the multi‐emulsion method of microfluidic chips can be used to enable the generation of microparticles with a core‐multi‐shell structure. These structures could potentially be used in a variety of medical applications by controlling the release mechanism of entrapped biomolecules. Using microfluidic chips for droplet generation, it is possible to control the size of microparticles through both continuous and dispersed phase flow rates to produce aerogel microparticles with desired diameters and size distribution. The microfluidic fabrication method of aerogel microparticles and hollow microspheres can be simply employed in various material systems by optimizing the flow rate, gelation time, temperature, and selection of continuous and dispersed phases. This production of microparticles and core‐shell hollow microspheres with controllable sizes enables aerogel materials for possible drug delivery applications.^[^ ^99^ ^]^

### 4.2 Micro‐Extrusion based 3D Printing

3D printing is a scalable and versatile technique for the fabrication of 3D aerogels made of various ink compositions, including silicon carbide (SiC) ceramics,^[^ ^133^ ^]^ carbon‐based materials,^[^ ^134^ ^]^ gold,^[^ ^135^ ^]^ cellulose,^[^ ^136^ ^]^ Kevlar,^[^ ^137^ ^]^ and SF.^[^ ^138^ ^]^ With the ability to develop complex architectural models at a faster rate than conventional approaches,^[^ ^139^ ^]^ 3D printing technology has paved the way for a variety of applications such as supercapacitors,^[^ ^140^ ^]^ electrodes,^[^ ^141^ ^]^ and tissue engineering.^[^ ^14^, ^142^ ^]^

Direct ink writing (DIW), otherwise called micro‐extrusion‐based 3D printing, is the most well‐known AM process for aerogels. Extrusion‐based DIW is a 3D printing method that produces structures with controlled composition by a continuous flow of a slurry of material (ink) from a nozzle to a substrate under specific pressure. DIW enables 3D printing of various materials, including polymers, metals, ceramics, and composites, in complex 3D shapes.

**Figure 9:**
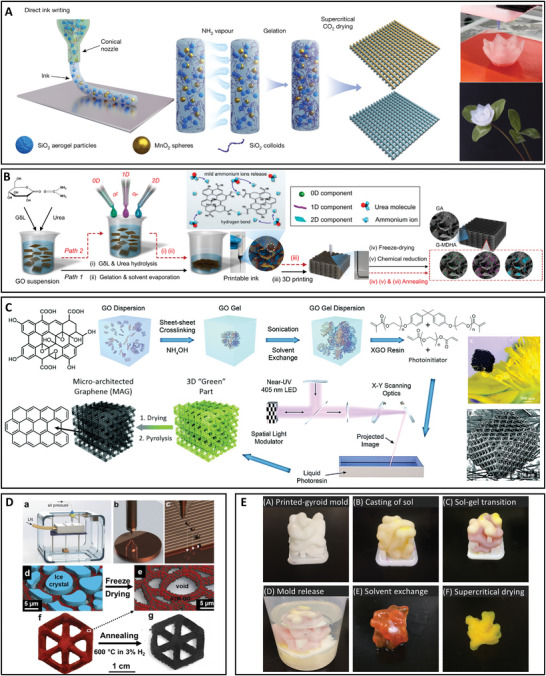
3D printing of aerogels. A) Schematic representation of direct ink writing (DIW) of silica aerogels. The images show a 3D‐printed flower of silica aerogel (38 layers). Reproduced with permission.^[^ ^12^ ^]^ Copyright 2020, Springer Nature. B) Direct 3D printing of hybrid aerogels with mixed‐dimensional graphene‐based additives. Reproduced with permission.^[^ ^145^ ^]^ Copyright 2018, American Chemical Society. C) Schematic shows resin development, micro‐stereolithography, drying, and pyrolysis processes for developing GAs. Optical and SEM images of pyrolyzed octet‐truss aerogels are represented. Reproduced with permission.^[^ ^151^ ^]^ Copyright 2018, Royal Society of Chemistry. D) Drop‐on‐demand (DoD) inkjet printing of MoS_2_/GAs. Reproduced with permission.^[^ ^157^ ^]^ Copyright 2019, Elsevier. E) Templated‐assisted fabrication of polyimide aerogels with complex geometries. Reproduced with permission.^[^ ^160^ ^]^ Copyright 2019, American Chemical Society.

3D printing of pure silica aerogels has been hampered by material drying issues that arise during the process. Zhao et al. introduced a novel technique for direct ink writing of pure silica aerogels (**Figure** **9**A).^[^ ^12^ ^]^ A 3D printable slurry was prepared by dispersing silica aerogel power in a 1‐pentanol‐based silica sol. By using low vapor pressure of pentanol, ink drying problems throughout the 3D printing process were reduced. The increased loading of gel particles improved the shear‐thinning behavior of the 3D printing process. The 3D‐printed silica sol structure was gelled by CO_2_SCD after exposure to ammonia vapor. In this way, aerogel structures of various complex shapes were fabricated with high precision and improved fidelity. In another study, to develop surface‐functionalized GAs, GAs were first 3D printed via DIW in GO ink, then freeze‐dried and annealed at 1050 °C.^[^ ^143^ ^]^ The 3D‐printed aerogels have open porous structures that can be functionalized. Therefore, surface functionalization was conducted through electrochemical oxidization using a calomel electrode at a potential of 1.9 V and 0.5 m KNO_3_ for 3 h. The large, functionalized pore area enabled high capacitance along with improved ion accessibility.

One of the major challenges in any extrusion‐based 3D printing technique is manipulating the gel viscosity and rheological performance. The ink composition defines the rheological properties and, consequently, the resolution of the printed construct. Inadequate rheological properties, such as low viscosity, can potentially reduce structural integrity, resulting in the failure of the printed structure. Also, if the ink has a high viscosity or contains a lot of additives/particles, the nozzle can become clogged. In addition, the shear‐thinning behavior of the designed ink is a key parameter in 3D printing. Tang et al. used direct 3D printing to fabricate the architecture of Ni_0.33_Co_0.66_S_2_/GA with an interconnected framework and an increased specific surface area of 212 m^2^ g^−1^.^[^ ^144^ ^]^ They added sodium alginate to the GO solutions and the novel precursor Ni_0.33_Co _0.66_(OH)_2·_ *x*H_2_O to improve viscoelastic behavior and printability. In other studies, various materials, including Ca^2+^ ions and urea, have been used to tune the rheological properties of the main ink to improve printability.^[^ ^134^, ^145^ ^]^

DIW technique can also be used to fabricate microlattices with precise control over the architecture and configuration. Microlattices are functional in many applications due to their lightweight, customizable high porosity, high elastic behavior, etc.^[^ ^65^, ^134^, ^146^ ^]^ Jiang et al. developed a 3D GA microlattice structure with high super capacitance using direct 3D printing.^[^ ^134^ ^]^ For this, Ca^2+^ ions were added to the GO‐based sol ink to obtain a 3D printable gel that can be printed at room temperature. The structures were 3D printed at a print speed of 10 mm s^−1^ using a nozzle with an internal diameter of 100 to 400 µm. Then, the freeze‐drying and hydroiodic acid reduction treatment was performed to obtain a graphene microlattice aerogel that converts GO into reduced GO (rGO)—adding more than 15×10^−3^ m Ca^2+^ ions resulted in rapid ion crosslinking and nozzle clogging issues.

Hybrid aerogels can be obtained by incorporating several functional additives into the ink.^[^ ^147^ ^]^ Yan et al. 3D printed nanocomposite aerogel microlattices composed of reduced GO/CNT (rGO/CNT) additives for a dendrite‐free anode.^[^ ^146^ ^]^ Tang et al. 3D printed graphene‐based aerogels using various dimensional additives (i.e., 2D+nD, n is 0, 1, or 2) as represented in Figure 9B.^[^ ^145^ ^]^ The major challenges in the 3D printing of hybrid ink compositions include making homogenous ink‐free agglomerated additives, manipulating the inks into desired complex shapes, and interconnecting 3D printing on curvy substrates. To adjust the rheological properties of the ink to be appropriate for 3D printing, urea was used for mild crosslinking of GO flakes, and the pH was tuned to control the crosslinking rate. In another study, He et al.^[^ ^148^ ^]^ proposed a carbon nitride‐based hybrid aerogel membrane fabricated by DIW for solar wastewater purification. A hybrid ink of g‐C_3_N_4_ nanosheets and sodium alginate (to improve rheological properties) was prepared and 3D printed. To demonstrate the versatility of the 3D printing method, the ink was 3D printed under three different conditions: 1) air, 2) a supporting reservoir of CaCl_2_/glycerol solution, and 3) a supporting reservoir of Pluronic F127.

Direct 3D printing can also be employed to fabricate multi‐material devices. Qian et al. used all‐printing methods for the facile fabrication of aerogel‐based triboelectric power sources for sensors.^[^ ^149^ ^]^ The nanogenerator DIW 3D printed with micropatterns was used to fabricate a micro‐nanoporous cellulose nanofiber (CNF) aerogel. First, the conductive Ag ink was 3D printed on two polyethylene terephthalate (PET) substrates as electrodes. Then, the CNF ink was 3D printed on one of the Ag/PET substrates and subject to freeze‐drying and annealing processes to obtain a hierarchical micro‐nanopatterned CNF aerogel. PDMS was also 3D printed on the second Ag/PET substrate. Finally, the PET/Ag/CNF aerogel layer was assembled with a PET/Ag/PDMS layer to form a sandwich nanogenerator device. Using this fabrication method, the output voltage was 175% compared to generators fabricated with molding techniques. The high throughput was attributed to the improved contact area, and the surface roughness was due to the architectural design with hierarchical micro‐nanoporosity.

### 4.3 Stereolithography (SLA) with Digital Light Processing (DLP)

SLA with DLP enables the fabrication of complex architectures at higher resolutions compared to extrusion‐based 3D printing technologies. The SLA process involves illuminating a UV laser into a vat of photocurable resin based on a predefined CAD model. After irradiating each layer with a laser, the next layer of resin is applied, and layer‐by‐layer printing continues. The DLP mechanism uses a similar printing concept but uses a digital projector instead of a UV laser to cure all points of each layer by exposing them to UV light simultaneously. Visible light sources of various wavelengths can be used for the 3D printing of visible light‐curable materials.^[^ ^150^ ^]^

Hensleigh et al. used projection micro‐stereolithography (PµSL) to fabricate complex GAs consisting of hierarchical stretch‐dominant micropatterns with high functional resolution on the order of a few microns (Figure 9C).^[^ ^151^ ^]^ They developed a photocurable GO resin that can rapidly solidify when exposed to near‐UV light with a wavelength of 450 nm and has a viscosity low enough to allow a layer‐by‐layer 3D printing process. The diluted resin consisted of 1 wt.% GO with 12 wt.% photocrosslinkable acrylates and 2–4 wt.% photoinitiator in N, N‐dimethylformamide solvent. After 3D printing, the green part was freeze‐dried, and pyrolysis of the structures removed most of the photopolymer and reduced GO to rGO. The use of crosslinked GO particles instead of pure GO flakes resulted in a larger surface area.

Through a 3D printing/lithography approach, Saeed et al. developed silica alcogels by free‐radical polymerization of a photocurable resin by irradiating a laser.^[^ ^152^ ^]^ The prepolymer was prepared by mixing tetraorthosilicate, trimethoxysilylpropyl methacrylate, and AlCl_3_. 6H_2_O in water‐ethanol azeotrope, and eosin Y and tertiary amine were used as photoinitiators and co‐initiators. The prepolymer was poured into a Petri dish and covered with a stencil mask. Then, a laser with a wavelength of 532 nm was illuminated onto the prepolymer through a hole in the mask. After polymerizing a thin layer of material, a certain amount of prepolymer was added to the Petri dish to form the next layer. This approach continued until the entire structure was formed. The rapid gelation of the layers upon laser illumination stems from the exothermic nature of alkoxide hydrolysis, which promotes a condensation process known as an endotherm. The polymerization thus generated heat, which aided in rapid gelation. The 3D printed structures were then dried under supercritical conditions to form low‐density aerogels.

### 4.4 Droplet Jetting 3D Printing

Droplet jetting is another 3D printing technique for developing engineered aerogels. Droplet jetting (also known as inkjet printing, ink jetting, or material jetting (MJ)) is considered one of the DIW methods. In this method, instead of extruding a continuous flow of material, droplets of photosensitive ink are dispensed onto the printed substrate. Then, the 3D‐printed structure is exposed to UV or visible light for layer‐by‐layer crosslinking. The continuous inkjet systems include a continuous flow of droplets that can be selectively deflected into the recycling system. High‐speed 3D printing is feasible with continuous inkjet systems. On the other hand, drop‐on‐demand (DoD) MJ systems utilize piezoelectric or thermoelectric elements to control droplet deposition directly.^[^ ^153^ ^]^ The newly developed DoD MJ processes use piezoelectric printheads capable of dispensing high‐viscosity inks.^[^ ^154^ ^]^ This method allows large or small pattern development by depositing the precise amount of ink on the substrate. Less material wastage in these methods lowers the cost of 3D‐printed components.^[^ ^155^ ^]^

Yan et al. developed a hierarchical silver nanowire aerogel using DoD inkjet printing integrated with a freeze‐casting method.^[^ ^156^ ^]^ In the proposed method, Ag nanowire ink mixed with DI water was prepared and dispensed droplet‐by‐droplet on a printing bed with a temperature of −30 °C. Since the temperature of the bed was much lower than the freezing temperature of the water, rapid nucleation of ice crystals occurred as soon as the droplets hit the printing bed. The frozen samples were kept at −70 °C for 1 day for further ice nucleation, followed by a sublimation process to form the final aerogel. Using this approach, the aerogel density was controlled and measured as low as 1.3 mg cm^−3^. The 3D structures composed of complex geometries with positive and negative Poisson ratios were prepared and characterized in electromechanical properties. Following a similar fabrication method, Brown et al. developed a hybrid aerogel for sodium‐ion batteries using ammonium thiomolybdate (MoS_2_) ink mixed with GO (Figure 9D).^[^ ^157^ ^]^ Subsequently, a 3D printing‐freeze casting process was implemented, followed by freeze‐drying and thermal annealing to convert GO to rGO, and developed a hybrid aerogel of porous rGO with MoS2 NPs immobilized on the surface. The interconnected rGO framework dramatically improved its electrical properties. In another study, Koo et al. 3D printed silica aerogel thin film patterns on a silicon wafer using a commercial DoD inkjet system as insulation for electronic devices and chips.^[^ ^158^ ^]^ A silica aerogel powder was dispersed in a mixture of DI water and DISPERBYK for 24 h to wet the additive and disperse it easily. Then, PVP‐k30 as a binder and 1,5‐pentadiol as a humectant was added and mixed for an additional 24 h. The inkjet printer cartridge was cleaned and filled with silica‐aerogel ink developed for the inkjet printing of patterns. The printed patterns were placed in an oven at 50 °C and dried for 24 h. The sample containing the dispersant showed much more uniform drying than the samples without the dispersant. Due to well‐distributed particles, the surface of the 3D‐printed pattern was smooth, and the thickness was well‐controlled. **Table** **4** compares various 3D printing techniques in terms of precursor, print speed, and resolution.

**Table 4:** Precursor materials, printing speed, and resolution of popular 3D printing techniques utilized for aerogel fabrication

3D printing technique	Precursor materials	Printing speed [mm s^−1^]	Resolution [µm]	Refs
FDM	Thermoplastic filaments such as ABS, PLA, polycarbonate, etc.	20–100	50–200	[161]
DIW	Any type of material that can be manipulated into a printable ink	1–20	100–610	[162]
SLA	A resin including photo‐active monomers	Depending on the SLA type (tens of millimeters per hour to thousands of millimeters per second)	20 µm or less (especially for µSLA)	[163]
Ink jetting/material jetting	A concentrated dispersion of particles in a liquid matrix (ink or paste)	5	5–200	[162, 164]
Piezoelectric‐pneumatic material jetting	A dispensable paste (even high viscose up to 40 000–50 000 mPa s at 10 s^−1^)	104	500–600	[162]

Since direct 3D printing of gels can be limited in terms of the complexity of the 3D printed structures, template‐assisted fabrication processes are common for fabricating complex architectural structures.^[^ ^159^ ^]^ Teo et al. introduced a sacrificial molding technique to develop complex double continuous aerogel structures (Figure 9E).^[^ ^160^ ^]^ Because high‐resolution 3D printing of thermoplastics is feasible with the fused deposition modeling (FDM) process, sacrificial hollow polystyrene molds were 3D printed using FDM. Then, the mold was filled with sol, the sol‐gel transition was implemented, and the sacrificial mold was removed from the organic solvent. The gel was dried at supercritical conditions to form an aerogel with a porosity of 98.8%. The structure showed high elasticity compared to the monolithic polyimide aerogel.

In conclusion, the exceptional designs of mesostructures can be efficiently made by 3D printing. This approach helps in the design of an application‐specific aerogel with tuned properties. Hence, despite the upcoming technological challenges, it is believed that the implementation of printing technologies could open the door to a new generation of affordable and customizable aerogels that could possibly create new functionalities and extend/enhance the existing applications, in particular, in biomedical fields. A comprehensive review of 3D‐printed aerogels can be found at [139] by Feng et al.

## 5 Biomedical Applications

**Figure 10:**
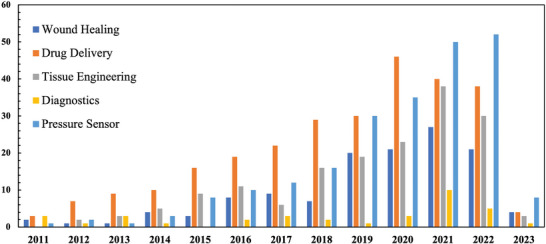
Number of publications in Web of Science Core Collection database for the search criteria “aerogel AND wound healing”, “aerogel AND drug delivery”, “aerogel AND tissue engineering”, “aerogel and diagnostics”, “aerogel and pressure sensor” (search date: Feb 19, 2023).

Porous biomaterials having interconnected pores and high specific surface areas are particularly interesting for biomedical applications, and aerogels can answer this challenge as lightweight materials. The search on the Web of Science, as shown below (**Figure** **10**), indicates the emerging interest in the aerogels’ application in the biomedical field.

### 5.1 Wound Healing

Wound healing is a highly orchestrated pathophysiological process that consists of numerous phases and a set of cell types, genes, and cytokines.^[^ ^165^ ^]^ Wound repair consists of three overlapping phases; inflammatory, proliferative, and remodeling.^[^ ^166^ ^]^ In the inflammatory phase, inflammatory cells migrate to the damaged area to clear the area of infection and establish a microenvironment for the wound healing process.^[^ ^167^ ^]^ In wounded tissue, white blood cells are responsible for removing damaged cells and blood clots and releasing cytokines and growth factors that trigger proliferation.^[^ ^168^ ^]^ During proliferation, cells grow into the wound, and new tissue progressively remodels the extracellular matrix (ECM).

Several conditions can negatively affect healing outcomes, including changes in pH, decreased oxygen levels, and bacterial growth in the wounded area.^[^ ^169^ ^]^ Due to the unique properties of aerogel‐based materials, including increased surface area, porosity, and permeability, they are highly effective in absorbing wound exudate. In this way, the aerogel‐based scaffold can provide appropriate conditions, such as moisture and pH control to the damaged site.^[^ ^10^ ^]^

Baldino et al. developed alginate, gelatin, and alginate‐gelatin aerogels via SCD.^[^ ^170^ ^]^ For alginate aerogels, 5% alginate was crosslinked in CaCl_2_ to promote gelation before water removal through multi‐step solvent exchange while increasing the ethanol concentration (10–100% v/v). For gelatin aerogels, a 5% gelatin solution was crosslinked in a coagulation bath of glutaraldehyde before water removal, following the same procedure for alginate. The alginate‐gelatin aerogel was produced with a combination of methods used for alginate and gelatin, respectively. The SCD method was performed at a flow rate of 1 kg h^−1^ for up to 8 h to remove the ethanol content and obtain alginate, gelatin, and alginate‐gelatin aerogels. Their results showed that the diffusion capacity of each gelatin and alginate hydrogel was preserved by the SCD process, preserving the nanostructure of the hydrogel in the corresponding aerogel. Recently, Franco et al. evaluated the application of novel material for wound healing through supercritical‐CO_2_ impregnation of mesoglycan with alginate aerogels.^[^ ^171^ ^]^ Mesoglycan is a mixture of glycosaminoglycans obtained from the mucosa of the porcine intestine. It is a promising bioactive substance for wound healing by promoting the migration and differentiation of keratinocytes.^[^ ^172^ ^]^ After the impregnation of mesoglycan in alginate aerogels, material‐cell interactions were assessed in vitro, including cytocompatibility and wound‐healing efficacy. Their results showed a 75% increase in covered distance (in vitro wound healing) after 24 h for keratinocytes treated with mesoglycan‐loaded alginate aerogels compared to untreated cells. However, it should be noted that the in vitro experiments were performed using an eluent of mesoglycan‐alginate aerogels without the material in direct contact with the cells. Therefore, further evaluation using an in vivo wound healing model is needed.

In a similar approach used to produce aerogels, Keil et al. evaluated Ca—Zn—Ag‐loaded alginate aerogels for potential applications in wound healing.^[^ ^173^ ^]^ The swelling behavior, antimicrobial properties, and release profile of Ca, Zn, and Ag were studied, and the results demonstrated a large ability to absorb fluids up to 3000% from exuding wounds and sustained release of the loaded metals. On the other hand, the antimicrobial effect of Ca—Zn—Ag‐loaded alginate aerogels was lower in contrast to the control AgNO_3_ (silver nitrate). In addition, analysis of the immunomodulatory function of Zn and cell culture studies showed high availability and anti‐inflammatory activity of the Zn species released from the alginate aerogels. In the same way, Raman et al. produced and evaluated alginate aerogels containing Zn and Ag.^[^ ^174^ ^]^ The authors reported that Zn release where Zn‐rich supernatants suppressed nitric oxide production in RAW 264.7 macrophages. However, there are no in vivo results demonstrating the wound healing properties of Ca—Zn—Ag‐loaded alginate aerogels in both previously mentioned studies.

Batista et al. developed aerogel fibers composed of alginate and CS. The authors assessed the ability of these aerogel fibers to induce wound healing in vitro.^[^ ^175^ ^]^ A W/O emulsion is made from an aqueous alginate solution (3% by weight, aqueous phase) to produce alginate‐CS fibers. The emulsion is then slowly added to the paraffin oil and Span 80 (3% by weight, oil phase) and stirred at 24 000 rpm for 2 min. The polyelectrolyte complex of the alginate and CS hydrogels was formulated by the emulsion‐gelation method. To this end, an acidic solution of CS (1.5 wt.%) is mixed with the alginate emulsion and stirred at 800 rpm for 60 min. In addition, CO_2_SCD was performed following alcogel conversion via solvent exchange (30–100% of ethanol). With similar results obtained by Baldino et al. for mesoglycan‐loaded alginate aerogels,^[^ ^170^ ^]^ alginate‐CS aerogel fibers were shown to be biocompatible. They also presented recovery of the scratch area of approximately 75% for the in vitro wound healing assays, which was significantly higher than the untreated control (≈50%).^[^ ^175^ ^]^ Besides, alginate‐CS aerogel fibers showed anti‐bacterial activity against *Staphylococcus aureus* and *Klebsiella pneumoniae*. These studies show that alginate‐based aerogels in monoliths or fibrous form can be generated by gelation methods followed by solvent exchange and CO_2_SCD. Moreover, various natural polymers, including gelatin and CS, can be incorporated to provide varying physicochemical properties.^[^ ^176^ ^]^ In addition to the drugs/chemicals, trace metals such as Zn and Ag can be loaded onto the solid backbone of the aerogel to add anti‐bacterial properties.

In another approach, Ko and Kim developed and evaluated a dual‐crosslinked CS aerogel as a future biomaterial for wound healing applications.^[^ ^177^ ^]^ By employing 1‐butyl‐3‐methylimidazolium chloride as the ionic solvent, the hydroxyl groups of CS are crosslinked through covalent bonds with epichlorohydrin. On the other hand, the amino group of CS is crosslinked through ionic interaction with itaconic acid. After crosslinking, dialysis was performed prior to freeze‐drying to obtain a dual‐crosslinked CS aerogel. The aerogel exhibits a complex porous arrangement and a swelling capacity of over 1.2 g g^−1^. Furthermore, the aerogels showed cytocompatibility in in vitro tests with human colon cancer cells (HT29). Piątkowski et al. used microwaves to synthesize CS aerogels loaded with gold NPs.^[^ ^178^ ^]^ Briefly, their production method consists of treating CS with NaOH (sodium hydroxide) solution at high temperatures (≈135 °C) for up to 30 min. The CS is then stirred in acetic acid (5%), and then gold NPs are added. After that, glutamic acid and aspartic acid are added, and 1,2‐propanediol is used as a high boiling point solvent and the crosslinking agent. Similar to the previously mentioned CS, alginate aerogel results, CS aerogel using microwave‐assisted method showed cytocompatibility in in vitro assays using L929 fibroblasts (mouse subcutaneous connective tissue, areola, and adipose).

**Figure 11:**
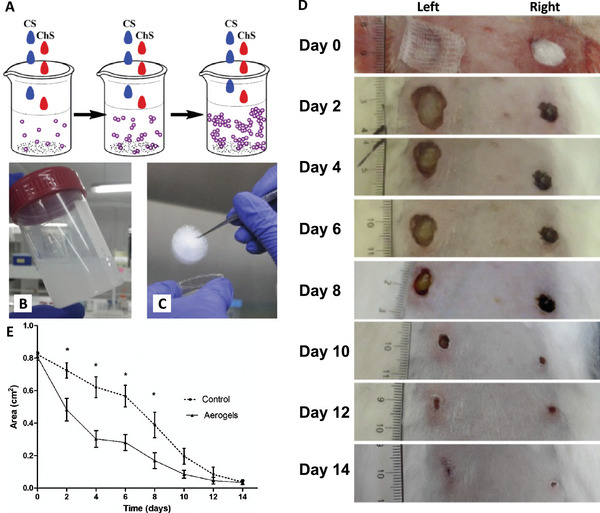
Fabrication and characterization of aerogel for wound healing application. A) Mixing steps for complexation; B,C) before and after freeze‐drying process. D) In vivo wound closure test on the back of rabbits treated with aerogels (right) and saline solution (left). E) Kinetics of wound closure. Reproduced with permission.^[^ ^179^ ^]^ Copyright 2018, Wiley.

Concha et al. performed wound healing efficacy of aerogels in vivo (in New Zealand rabbits).^[^ ^179^ ^]^ In their work, aerogels were produced by combining CS and chondroitin sulfate (ChS). As such, the blend of the two materials as a colloidal suspension was freeze‐dried (**Figure** **11**). Their results showed faster‐wound closure during the first 8 days after surgery compared to the untreated wounds. In both cases, aerogel‐treated and untreated wounds closed completely on day 14. The most significant difference between aerogel‐treated and untreated wounds was observed on day 4. This provides a clue that aerogel can improve the early stage of wound healing. In addition to improving wound healing, the additional antibacterial properties of CS may help prevent wound infection.

**Figure 12:**
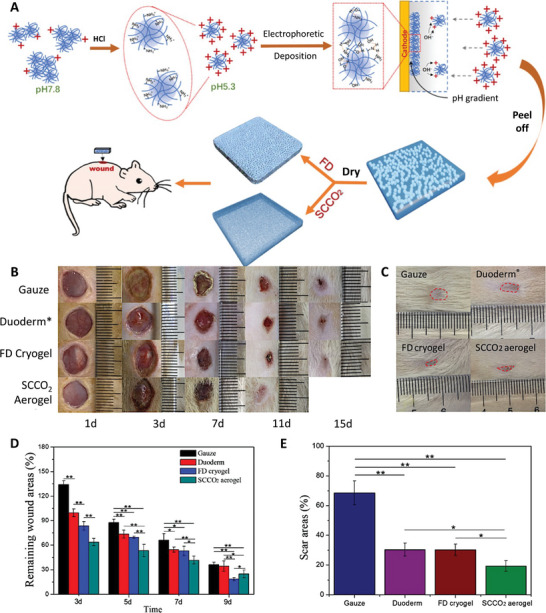
A) Schematic demonstration of electrophoretic deposition of chitin NPs and the obtained aerogels for wound dressing. B) Wound healing progress of the tissue when treated by the Gauze, DuoDERM (a commercial wound dressing), freeze‐dried cryogel, and CO_2_SCD (SCCO_2_) aerogel groups. C) Wounded area 17 days after surgery. D) Assessment of wound closure over time after surgery. E) Assessment of the area of scars. Reproduced with permission.^[^ ^180^ ^]^ Copyright 2019, American Chemical Society.

Guo et al. established a technique for producing hierarchically organized aerogels.^[^ ^180^ ^]^ This was achieved with the aid of electrically assisted assembly of chitin NPs that can grow thick and form free‐standing hydrogel structures in the fabrication of aerogels and cryogels for wound healing applications (**Figure** **12**). To this end, the pH of 0.7 wt. % chitin NP suspension was adjusted to pH 5.3, and hydrogen peroxide (H_2_O_2_) (0.1 wt. % final concentration) was added to the NP suspension. Then, titanium and platinum plates were used as cathode and anode, respectively. The cathode and anode were immersed in the NP suspension before applying a constant current of 1.5–2.5 A m^−2^ for 60 min to induce chitin NP deposition. The resulting chitin hydrogel was dried with CO_2_SCD or freeze‐drying to obtain chitin aerogel and cryogel. The wound‐healing properties of the aerogel were evaluated in vivo in a full‐thickness skin defect model. Their results suggest that wound healing is mostly improved during the first 9 days, suppressing inflammation and granulation tissue formation. While the freeze‐dried cryogels were not significantly different from the commercially available control, the aerogels generated via CO_2_SCD showed enhanced wound healing properties. Furthermore, the chitin‐based aerogel promoted macrophage migration, fibroblast proliferation, granulation, and vascularization within the treated wound, and the scar area was also smaller than that of the untreated group. The works performed by Concha et al.^[^ ^179^ ^]^ and Guo et al.^[^ ^180^ ^]^ demonstrate the importance of in vivo evaluation of aerogels to be developed for wound healing applications. Several examples of the use of aerogel for wound healing applications are listed in **Table** **5**.

**Table 5:** List of fabricated aerogels for wound healing applications with respect to their porosity characteristics

Material	Sol‐gel Approach	Drying Approach	Porosity [%]	Pore Size	Pore Volume [cm^3^ g^−1^]	Surface Area [m^2^ g^−1^]	Mechanical Property [MPa]	Cell type	In vivo	Ref.
Alginate/gelatin	Bath of CaCl_2_	CO_2_SCD	90	100 nm	–	248	Y'sM: 0.78 ± 0.06 TSB: 2.33 ± 0.25 MPa	–	–	[170]
Alginate	Bath of CaCl_2_	CO_2_SCD	85	100 nm	–	–	–	Fibroblasts and Keratinocytes	–	[171]
Alginate	Ca^2+^ Zn^2+^ and Ag^+^	CO_2_SCD	–	–	–	–	–	Macrophages	–	[173, 174]
Alginate/chitosan	Chitosan	CO_2_SCD	–	–	1.41–2.49	162–302	–	Fibroblasts	–	[175]
Chitosan	Covalent: epichlorohydrin Ionic: Itaconic acid	Freeze‐drying	–	–	–	–	–	Colon cancer cells (HT29)	–	[177]
Chitosan	1,2‐propanediol	microwave reactor	>90	300 – 500 µm	–	–	Y'sM: 30 MPA TSB: 4 MPa	Fibroblasts	–	[178]
Chitosan	pH‐assisted ionization	Freeze‐drying	>99	–	–	–	–	Fibroblasts	New Zealand rabbits	[179]
Chitin NPs	electroassembly	CO_2_SCD and Freeze‐drying	87 97	100 nm 2 – 4 µm	1.94 0.10	289 17	Y'sM: 1.3 and 0.38	Fibroblasts	Sprague‐Dawley rat	[180]

^a)^ Y'sM: Young's Modulus, TSB: Tensile strength at break

### 5.2 Drug Delivery

There are two main routes of drug delivery that are commonly used: local or systemic. Systemic drug delivery can be further subdivided into enteral (i.e., oral, sublingual, vaginal, and rectal) or parenteral (i.e., intravenous, inhalation, and transdermal) delivery.^[^ ^181^ ^]^ Each delivery method has its own advantages and disadvantages. For example, the oral route is easy and preferred by most patients. However, most orally administered drugs may exhibit unpredictable absorption kinetics due to gastric acid and enzymatic degradation. In comparison, the inhalation route may be a better alternative because of the large surface areas of the respiratory endothelium that allows for rapid drug absorption. However, the bioavailability of a drug via the inhalation route relies on the inhaler mechanism and drug particle size. In addition to the route of administration, other factors that significantly influence the drug loading and release profile include the drug's molecular structure, solubility (i.e., hydrophilic vs. hydrophobic), initial concentration, and drug delivery scaffold system.

Recently, interest in the use of aerogels as drug carriers has increased significantly^[^ ^182^ ^]^ due to promising properties, such as large pore volume (≈7 m^3^ g^−1^), high porosity (50–99.8%), high specific surface area (>2000 m^2^ g^−1^), and low density (≈0.05 g cm^−3^) for loading active compounds.^[^ ^8^, ^183^ ^]^ The pore sizes of aerogel can also be adjusted,^[^ ^184^ ^]^ protecting it from rapid degradation and making it an excellent carrier suitable for the storage of diverse drugs (i.e., Ibuprofen (IBU),^[^ ^185^ ^]^ Ketoprofen (KET),^[^ ^186^ ^]^ Triflusal,^[^ ^187^ ^]^ Dexpanthenol,^[^ ^188^ ^]^ Nicotinic acid,^[^ ^189^ ^]^), thereby increasing the stability of the drug.^[^ ^190^ ^]^ Aerogel nanoscale pores not only serve as a drug matrix that can load large amounts of drugs with rapid water absorption but also accelerate drug release. These properties demonstrate the potential for effective use as drug carriers.^[^ ^9^ ^]^ Based on their unique characteristics mentioned above, aerogels can provide essential insights into drug delivery to eliminate the effects of low water solubility and toxicity of drugs.^[^ ^8^ ^]^ Therefore, the most recent studies focused on the use of aerogels in drug delivery applications are discussed in the following sections.

Many drugs can be incorporated into the aerogel matrix by either CO_2_SCD impregnation^[^ ^185^, ^191^ ^]^ or during the sol‐gel process.^[^ ^192^ ^]^ This process mainly depends on the stability of the drug and its affinity with the aerogel matrix or solvents (i.e., water, CO_2_SCD, etc.) in the solvent exchange process. Changes in process parameters such as the gelation time, initial precursors, catalyst, rate of solvation, gel processing, and gelation conditions can enable aerogels with various pore sizes and porous morphologies. Also, the drug release profiles of the loaded aerogel are highly dependent on the intrinsic properties of the aerogel.^[^ ^9^ ^]^ It can be altered by using techniques such as multi‐membrane hydrogel formation, surface derivatization, and post‐coating of the aerogel to achieve a specific release profile (i.e., sustained, controlled, or bolus release).^[^ ^193^ ^]^

Various types of aerogels (i.e., organic, inorganic, composite, and surface‐functionalized aerogels) have previously been utilized as carriers for DDS.^[^ ^8^, ^194^ ^]^ Studies focusing on inorganic aerogels with DDS have mainly focused on the use of silica‐based aerogels to investigate their structural and morphological properties (i.e., pore size, density, specific surface area) to control drug adsorption and drug‐loading capacity.^[^ ^2^, ^195^ ^]^ In drug release studies, when compared to crystalline drug forms, hydrophilic silica aerogel exhibited higher drug loading and increased drug dissolution rate. Besides, drug stability during storage improved when incorporated into silica aerogel compared to other polymeric drug carriers.^[^ ^187^ ^]^ Silica‐based aerogels with drugs can also be tailored to enable bolus or sustained drug release, depending on the surface properties (hydrophobic or hydrophilic) of the silica aerogel. Drug loading can be achieved by incorporating hydrophobic drugs into hydrophilic aerogels or by loading hydrophilic drugs into hydrophobic aerogels.^[^ ^2^, ^183^ ^]^

In addition to inorganic aerogels, organic aerogels such as natural polysaccharides have also become promising candidates as drug delivery carriers.^[^ ^191^ ^]^ They can eliminate the limitations of inorganic aerogels, such as biodegradability while exhibiting properties similar to their silica‐based counterparts. Organic aerogels made from polysaccharides (i.e., CS) are of particular interest due to their biocompatible, biodegradable, non‐toxic, mucoadhesive, and high charge density properties. CS increases the solubility of poorly soluble drugs and has been shown to have a significant effect on fat metabolism.^[^ ^196^ ^]^ In addition, CS can help improve bioavailability, and its mucoadhesive properties can be further utilized to enhance drug absorption by extending the residence time of DDS on mucosal surfaces.^[^ ^197^ ^]^

Most polysaccharide‐based aerogels are mucoadhesive, making them the DDS of choice for oral/mucosal‐based drug delivery. This property helps increase the absorption of drugs by prolonging the contact time with the gastrointestinal mucosa. In 2015, García‐González et al. prepared starch, pectin, and alginate‐based aerogel microspheres and loaded anti‐inflammatory drugs, KET, and benzoic acid using CO_2_SCD‐assisted adsorption.^[^ ^46^ ^]^ Specifically for starch microspheres, the authors reported drug loadings of 1.0×10^−3^ and 1.7×10^−3^ g m^−2^ for KET and benzoic acid, respectively. The drug release kinetics of the two drugs were fitted to a first‐order model in which each drug has a different release rate constant. On the other hand, pectin and alginate aerogel microspheres had drug loadings in the range of 11–24 wt.% and were fitted to a Gallagher–Corrigan release model. The KET release from pectin and alginate microspheres was pH‐dependent. Based on these results, the authors suggest that drug loading and drug release profiles can be tuned by appropriately selecting the type of polysaccharide (i.e., starch, pectin, or alginate) used to prepare the aerogel.^[^ ^46^ ^]^

Gonçalves et al. formulated aerogel microparticles utilizing alginate (<50 µm) with a high specific surface area (370–548 m^2^ g^−1^) for oral/mucosal delivery of KET and quercetin (a plant flavanol).^[^ ^198^ ^]^ The hybrid aerogels were prepared by co‐gelling alginate with low methoxyl pectin or *κ*‐carrageenan. KET and quercetin were loaded into these microparticles with drug loadings of 17–22 wt.% and 3.1–5.4 wt.%, respectively. KET was loaded via absorption in CO_2_SCD_,_ whereas quercetin was loaded using supercritical anti‐solvent precipitation. Both drugs, KET and quercetin, were in an amorphous state after loading. Of note, Gonçalves et al. reported that alginate/*κ*‐carrageenan hybrid aerogel microparticles released both drugs slightly faster than alginate or alginate/pectin hybrid microspheres.^[^ ^198^ ^]^

Aerogels made of CNF have been reported to be effective gastro retentive DDSs for the tested model drug bendamustine HCl (a chemotherapeutic drug).^[^ ^199^ ^]^ Drug loading was performed via physical adsorption with 19% encapsulation efficiency. Bhandari et al. reported cumulative drug release over 24 h of 70% and 78% at pH 1.2 and 7.4, respectively. More importantly, a 3.25‐fold increase in drug bioavailability was observed in in vivo studies.^[^ ^199^ ^]^

Aerogel beads made of Fe(III)‐crosslinked alginate (average size of 3–5 mm) were loaded with the anti‐inflammatory agent IBU by an adsorptive deposition mechanism in a CO_2_SCD process.^[^ ^200^ ^]^ The release of IBU from the aerogel beads made of Fe(III)‐alginate was observed to be significantly faster when processed at neutral pH (7.4) compared to acidic pH (2.0). Veres et al. attributed this difference to an increase in the higher increased swelling rate of the alginate network at neutral pH, which results in faster dissolution. In another study, both IBU and ascorbic acid were loaded into the aerogel, and drug release was accelerated at both neutral and acidic pH. The authors hypothesize that the crosslinking agent Fe(III) is reduced to Fe(II) by ascorbic acid in an aqueous environment, which also prevents strong interactions with alginate. As a result, the alginate carrier matrix becomes hydrated, resulting in erosion and dissolution of the aerogel.^[^ ^200^ ^]^

Eleftheriadis et al. prepared mesoporous cellulose aerogels with pore sizes of 10 or 20 nm. They later passively loaded the cellulose aerogels with IBU and achieved a drug loading of 18.2 wt.% for aerogels with 10 nm pore size and 22.9 wt.% for aerogels with 20 nm pore size.^[^ ^201^ ^]^ The authors performed drug release studies in simulated intestinal fluids reflecting both fasting (pH 5.0) and fed (pH 6.5) states. In the study, the release of IBU was strongly pH‐dependent, resulting in a faster release at pH 5.0 than the longer‐term release at pH 6.5.^[^ ^201^ ^]^

Salbutamol is a bronchodilator and is used to treat bronchospasms. Obaidat et al. prepared CS aerogel microparticles using both SCD or freeze‐drying and observed varying physical properties, such as particle size and density, between the two drying methods.^[^ ^202^ ^]^ The microparticles were fabricated using SCD and were found to have densities ranging from 0.07 – 0.32 g mL^−1^ and particle sizes ranging from 8.1 – 238.95 µm. On the other hand, freeze‐dried microparticles have a tap density in the range of 0.19 – 0.27 g mL^−1^ and an average particle size of about 64 µm. The authors also observed that several aspects, such as molecular weight, polymer concentration of CS, and tripolyphosphate concentration, significantly affect the drug release profile of salbutamol and that 80–90% of the drug can be released within 4.5 h.^[^ ^202^ ^]^ However, it should be noted that the authors did not address the drug loading or encapsulation efficiency of the CS aerogel microparticles, making it difficult to evaluate whether these aerogel microparticles are effective for pulmonary drug delivery.

As mentioned above, drug solubility plays an important role in drug loading and release from aerogels. In a previous study, polyethyleneimine‐grafted CNF (CNFs‐PEI) were prepared and loaded with water‐soluble sodium salicylate (NaSA) with a significant drug loading capacity of nearly 287.39 mg g^−1^.^[^ ^203^ ^]^ They observed a pseudo‐second‐order release profile of the drug, a sustained and controlled release. A pH‐ and temperature‐dependent drug release was also reported, with a higher release observed with increasing pH from 2 to 7.4 and increasing temperature from 20 to 50 °C.

In general, the design of DDSs for hydrophilic or water‐soluble drugs is less complex than for hydrophobic or low water‐soluble drugs. Therefore, most of the research effort is devoted to improving hydrophobic drug loading and tuning release profiles. Nonsteroidal anti‐inflammatory drugs (NSAIDs), like IBU, KET, nimesulide, and indomethacin, are low water‐soluble drugs known to reduce pain, fever, and inflammation.

Obaidat et al. prepared carrageenan aerogel microparticles using the emulsion‐gelation technique and loaded IBU into microparticles using CO_2_SCD. They achieved drug loadings of 35–70 wt.%, depending on the type of carrageenan and crosslinker used.^[^ ^204^ ^]^ Veres et al. achieved 24 wt.% IBU loading and 14 wt.% KET loading in aerogels made with silica‐gelatin (3 wt.% gelatin content) and observed tenfold faster drug release in aerogels loaded with IBU and KET compared to pure silica aerogel.^[^ ^205^ ^]^ The authors suggest that the silica‐gelatin hybrid aerogels (SGHA) have stronger hydration compared to silica aerogels. This results in rapid desorption, as well as dissolution of drugs, IBU and KET through a hybrid matrix, compared to silica aerogels.^[^ ^205^ ^]^ In another study, Veres et al. prepared an array of 14 hybrid aerogels using silica and gelatin. Each aerogel was functionalized with one of three moieties: phenyl, C16 hydrocarbon chain, or methyl.^[^ ^206^ ^]^ Significant amounts of IBU and KET were loaded into the hybrid aerogels in the range of 15–25 wt.% and 10–15 wt.%, respectively.^[^ ^206^ ^]^ Silica aerogels with various densities (i.e., 0.033, 0.08, and 0.24 g cm^−3^) were prepared by Mohammadian et al. for KET loading and release.^[^ ^207^ ^]^ Compared to the crystalline form of KET, the authors observed increased KET release in silica aerogels, with the lowest density of aerogel exhibiting the highest amount of drug release.^[^ ^207^ ^]^

Lovskaya and Menshutina prepared alginate‐based aerogels using a dripping method to prepare particles, followed by CO_2_SCD to prepare the final porous aerogel particles.^[^ ^208^ ^]^ In addition to KET and nimesulide, the authors explored the loading and release of another low water‐soluble drug, loratadine (an antihistamine). Aerogels made of alginate were impregnated with KET, nimesulide, and loratadine at drug loadings of 18–29, 5–15, and 24–31 wt.%, respectively. The alginate aerogels were able to release the drugs approximately 6.6 times faster when compared to the release profile of the drugs alone.^[^ ^208^ ^]^

Shao et al. prepared alginate‐g‐P(NIPAM‐co‐NHMAM) aerogels with both temperature‐ and pH‐responsive properties as DDS for the NSAID indomethacin. They grafted alginate with N‐isopropylacrylamide (NIPAM) and N‐hydroxylmethylacrylamide (NHMAM) to achieve a drug loading efficiency of 13.24%.^[^ ^209^ ^]^ By fine‐tuning the ratio of NIPAM and NHMAM in the grafted aerogel, the authors reported that the lower critical solution temperature (LCST) of the aerogel could be adjusted between 27.6–42.4 °C. Above LCST, they also observed high responsiveness to both temperature and pH. For instance, hybrid aerogels have been reported to have faster drug release than alginate aerogels. However, it should be noted that an obstructed release of indomethacin was observed at acidic pH, whereas complete drug release was observed at neutral pH. Due to the temperature‐ and pH‐dependent drug release, the authors indicate the potential use of these hybrid aerogels in hyperthermia (high body temperature) and intestinal (low pH environment) treatment.^[^ ^209^ ^]^ In a study by Horvat et al., the authors coated medical‐grade stainless steel substrates with high methoxyl pectin‐xanthan aerogels using ethanol‐induced gelation and subsequent SCD. Indomethacin or diclofenac sodium was loaded into the aerogel to apply pain reduction and local inflammation prevention during total hip arthroplasty.^[^ ^210^ ^]^ All aerogel‐coated samples showed excellent corrosion resistance. The authors achieved a 4.2% indomethacin loading and 4.5% diclofenac sodium loading in the coating with the complete drug release after 24 h.^[^ ^210^ ^]^

Curcumin is the main active ingredient in the spice turmeric and is commonly used to treat conditions associated with pain and inflammation. However, due to its crystalline structure, it is poorly absorbed and therefore has low bioavailability. Ubeyitogullari and Ciftci prepared curcumin NPs with low crystallinity with an average size of 66 nm impregnated in nanoporous starch aerogel (NSA) with the help of CO_2_SCD.^[^ ^211^ ^]^ The authors reported a high encapsulation capacity of 224.2 mg g^−1^ NSA and increased solubility in digestive fluids, and improved bioaccessibility (173‐fold).^[^ ^211^ ^]^ Pantic et al. designed a DDS comprised of a pectin aerogel core containing curcumin with an external coating layer of CS.^[^ ^212^ ^]^ The rationale for this design was to use a CS coating layer to delay the dissolution of the pectin aerogel core, thereby delaying the subsequent release of the drugs. The authors showed that pectin aerogels achieved a burst release within 3 h, whereas the pectin aerogel coated with CS showed controlled release over 24 h.^[^ ^212^ ^]^

Paukkonen et al. investigated the drug release of three small molecules (MW < 500 g mol^−1^): metronidazole (MZ), nadolol (NAD), and KET; and three high MW proteins: 4 kDa Fluorescein isothiocyanate (FITC)‐dextran, bovine serum albumin (BSA), and lysozyme. These molecules were chosen due to their weight, size, and charge at neutral pH.^[^ ^213^ ^]^ The authors prepared anionic nanofibrillar cellulose (ANFC) aerogels using trehalose and PEG6000 as cryoprotectants. They observed that the higher the ANFC fiber content, the smaller the diffusion coefficients for the larger proteins. They concluded that in this DDS, the cationic charge of a molecule was a more important determinant than its size during diffusion. In comparison, for smaller molecules, the availability of free water had a more significant effect on diffusion than the ANFC fiber content, which only moderately affected drug release.^[^ ^213^ ^]^

**Figure 13:**
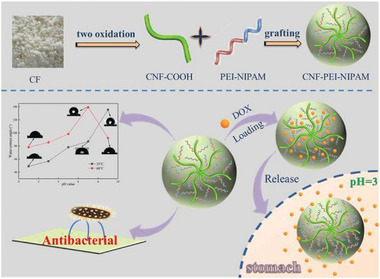
Smart CNFs as a sustained drug delivery platform for DOX via pH and temperature dual‐responsive release mechanism. Reproduced with permission.^[^ ^214^ ^]^ Copyright 2020, Elsevier.

Chemotherapy uses one or more anti‐cancer drugs to treat cancer. Unfortunately, most chemotherapeutic drugs are hydrophobic and have very poor solubility in water. Doxorubicin (DOX) is one such example. As shown in **Figure** **13**, Liang et al. prepared an aerogel by grafting a temperature‐ and pH‐responsive polymer, polyethyleneimine–N‐isopropylacrylamide (PEI‐NIPAM), onto CNF‐COOH.^[^ ^214^ ^]^ The resulting aerogel, CNF‐PEI‐NIPAM, enabled a significantly higher DOX loading capacity of 330.12 mg g^−1^ at pH 3 and temperature of 37 °C and a cumulative drug release rate of 59.45%. The authors also reported that the rate of drug release decreased as the temperature decreased from 37 to 25 °C, and the pH increased from 3 to 7.4.^[^ ^214^ ^]^

Fluorouracil (5‐FU) is a chemotherapeutic agent used to treat many solid tumors. Wang et al. prepared hybrid aerogels made of CS, carboxymethylcellulose, and GO using Ca^2+^ as a crosslinker. This hybrid aerogel had a 5‐FU drug loading efficiency of 54 wt.%.^[^ ^215^ ^]^ These hybrid aerogels showed pH‐dependent swelling and release behavior; acidic pH resulted in a swelling ratio (SR) of 2.23% and cumulative drug release of 26%, whereas neutral pH resulted in an SR of 20% and cumulative release of 68%.^[^ ^215^ ^]^ In another study, hybrid aerogels made of dopamine‐functionalized carboxyl‐functionalized MWCNT and konjac glucomannan were investigated for pH‐sensitive 5‐FU drug release in the aerogel matrix.^[^ ^216^ ^]^ The authors observed 48% and 62% 5‐FU drug release from the aerogel at pH 1.2 and 6.4 after 11 h, respectively.^[^ ^216^ ^]^

Methotrexate (MTX) is another chemotherapeutic and immunosuppressive drug used to treat blood, bone, lung, breast, head, and neck cancers. Nagy et al. prepared SGM through sol‐gel technique and co‐gelation and covalently linked MTX to the aerogel backbone by amide bonds.^[^ ^217^ ^]^ A drug loading efficiency was 6 wt.%. It should be noted that MTX could not be cleaved or delivered from the SGM aerogel particles in the buffer for 72 h. However, when tumor cells are present, SGHA particles produce free MTX and induce cytotoxicity against cell lines associated with the collagenase activity of the cells.^[^ ^217^ ^]^ Thus, the conjugation of MTX to the aerogel backbone provides a controlled release system.

Follmann et al. achieved high entrapment (98%) of the anti‐cancer drug camptothecin (CPT) with a sustained release over 2 weeks.^[^ ^218^ ^]^ The authors prepared a hybrid aerogel of a) KCC‐1 nanofibrous silica microparticles functionalized using CH_3_ groups, b) PVA, and c) polyacrylic acid (PAA), and crosslinked via solid‐state reaction. They observed that CPT release could be modulated by changing the ratio of PAA, PVA, and CH_3_‐modified KCC‐1.^[^ ^218^ ^]^ But more importantly, the aerogels are biocompatible with epithelial cells, fibroblasts, and kidney cells but induce cytotoxic effects on HeLa (HPV18‐positive), SiHa (HPV16‐positive), and C33A (HPV‐negative) cancer cells.^[^ ^218^ ^]^ Another important chemotherapeutic agent is paclitaxel (PTX), which treats lung, ovarian, and breast cancer. PTX does not provide oral bioavailability due to its low solubility in aqueous media, inadequate permeability in the intestine, and high levels of P‐glycoprotein efflux.^[^ ^219^ ^]^ To circumvent this issue, Wang et al. designed a nanoporous silica aerogel DDS for oral delivery of PTX and reported increased bioavailability as well as reduced side effects.^[^ ^219^ ^]^

**Figure 14:**
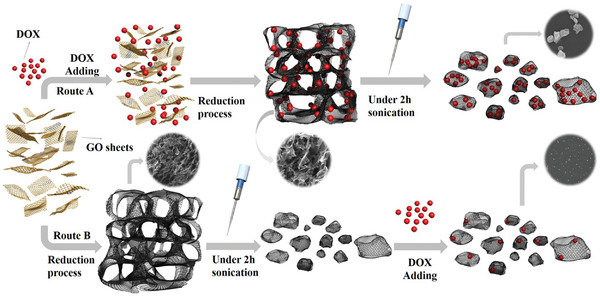
Schematic illustrating the fabrication of GA using GO sheets and demonstration of the drug loading steps (the in situ method (route A)) and the post‐synthesis of GA (step by step method (route B)). Reproduced with permission.^[^ ^220^ ^]^ Copyright 2020, Elsevier.

Recently, Ayazi et al. synthesized GA‐NPs (**Figure** **14**) and tested the loading and release profile of ionized drugs, such as amikacin sulfate, DOX, and d‐glucosamine hydrochloride, as well as non‐ionized drugs, such as PTX.^[^ ^220^ ^]^ They observed that DOX (with a partial positive charge) had a loading efficiency three times higher than that of PTX, but both had aromatic ring structures. Similarly, amikacin sulfate and d‐glucosamine hydrochloride also showed higher loading efficiency than PTX due to the positive partial charge. This led the authors to conclude that the electrostatic interaction between the ionized drugs and GO via hydrogen bonding is the dominant factor affecting the drug loading of GA‐NPs. Furthermore, DOX‐loaded GA‐NPs showed high pH‐sensitive release, with 60% being released within the first 24 h and the remainder after 5 days, indicating the advantage of targeting tumor cells.^[^ ^220^ ^]^

Mesoporous starch (MPS) aerogels are highly porous biomaterials and have been used for the delivery of genistein, phytoestrogen isoflavone, and chemotherapeutic drugs for the treatment of prostate, bladder, and breast cancer. Soleimanpour et al. suggest that the source of starch, the concentration of starch, the concentration of the ethanol used for solvent exchange, and the drying method all significantly affect the physical characteristics of the MPS aerogels.^[^ ^221^ ^]^ MPS particles with an average pore size of 105.4 nm were reported to be prepared using the following parameter combination: 14 wt.% corn starch and 100% ethanol concentration in the aging step, followed by rotary VD. The authors reported a high loading capacity (≈45%) and efficiency (≈80%) for genistein encapsulated in the MPS microstructure in a 1:1 weight ratio with enhanced dissolution.^[^ ^221^ ^]^

In addition to chemotherapeutic agents, antibacterial and biocide agents are other important drugs of great interest to researchers, especially in the treatment of infections at wound sites. Broad‐spectrum antibiotics such as vancomycin, doxycycline, and amoxicillin are particularly important because they strongly affect gram‐positive and negative bacteria. López‐Iglesias et al. prepared CS aerogel beads with a high porosity of 96% or more and a large surface area of 200 m^2^ g^−1^ or more. These aerogel beads were loaded with vancomycin to treat and prevent infection and effectively prevented *S. aureus* growth at the wound site.^[^ ^222^ ^]^ The authors observed a rapid burst release of vancomycin within the first hour, followed by a slow release, eventually remaining stable within 24 h. De Cicco et al. prepared aerogel microcapsules with an amidated pectin core containing doxycycline and a shell made of alginate with high mannuronic content.^[^ ^223^ ^]^ The authors reported a high encapsulation efficiency (87%) of doxycycline, extending drug release for up to 48 h. The release of doxycycline from the spherical particles depended on the drug/pectin ratio as well as alginate concentration; higher alginate in the outer layer delayed the drug diffusion from the expanded polymer matrix.^[^ ^223^ ^]^ Ye et al. prepared cellulose aerogels and loaded amoxicillin with a loading range of 4 to 13.45 µg cm^−2^.^[^ ^224^ ^]^ The authors demonstrated controlled drug release with burst release during the first couple hours, sustained drug release for 12 h, and showed excellent dose‐dependent effects against *E. coli, C. albicans, B. subtilis*, and *S. aureus*.^[^ ^224^ ^]^ In 2018, Silvetti et al. prepared fenugreek gum aerogels and loaded them with a mixture of two microbiocides: 5‐chloro‐2‐methyl‐4‐isothiazolin‐3‐one (CIT) and 2‐methyl‐4‐isothiazolin‐3‐one (MIT).^[^ ^225^ ^]^ A slow and complete release of CIT and MIT was achieved in water with the same minimal inhibitory concentration of free biocide, demonstrating activity against *S. marcescens and P. aeruginosa*.^[^ ^225^ ^]^

Rossi et al. prepared and evaluated an aerogel based on laccase‐oxidized galactomannans as DDS of lysozyme, an anti‐microbial enzyme that catalyzes cell wall destruction of certain bacteria.^[^ ^226^ ^]^ Lyophilization was used to prepare the aerogel, resulting in a water‐insoluble aerogel with a swelling rate of 20 times its initial weight in aqueous and organic solvents. The authors reported that lysozyme is retained and released in its active form, regardless of whether lysozyme was added before or after aerogel formation. This was demonstrated by testing the hydrolytic glycosidase activity of lysozyme release on cell wall peptidoglycans of lyophilized *Micrococcus lysodeikticus*.^[^ ^226^ ^]^

Certain wound dressing applications often require dual‐action devices to prevent infection while reducing pain. To this end, Follmann et al. prepared a novel hybrid aerogel by encapsulating mesoporous silica NP (SiNP) dispersed within a PVA/PAA hyperbranched polymer network.^[^ ^227^ ^]^ The anti‐inflammatory drug dexamethasone (DEX) was used as the model drug. The authors achieved a loading efficiency higher than 75%, with sustained drug release over 7 weeks or more. They also observed that the release pattern of DEX in the aerogels could be tailored by changing the weight ratio of PVA:PAA in the precursors. Furthermore, the aerogels exhibited bactericidal activity due to the SiNP functionalized with quaternary groups diffused into the aerogel.^[^ ^227^ ^]^

Hybrid aerogels have also been recently exploited to benefit from different physical behaviors and functions in drug delivery applications. Afrashi et al. developed a multi‐layer composite (MLC) consisting of a PVA substrate and silica aerogel powder packed with the anti‐fungal agent fluconazole and used it as a DDS to enable controlled release.^[^ ^228^ ^]^ Silica aerogel showed a higher release rate when compared to the release profiles of pure fluconazole, mainly due to its high surface area (>800 m^2^g^−1^) and porosity (>80%). PVA nanofibers were also observed to control the drug release rate, and in vitro tests further showed that the fluconazole‐loaded MLC had excellent anti‐fungal properties. Furthermore, the MLC containing silica aerogel with hydrophilic properties had a greater effect on the controlled release aspect when compared to the samples with hydrophobic properties. This property reduces the effect of the polymer coating on the aerogel due to the poor adhesion of the aerogel to the polymer surface, eventually resulting in the separation of the aerogel powder from the sample.

**Figure 15:**
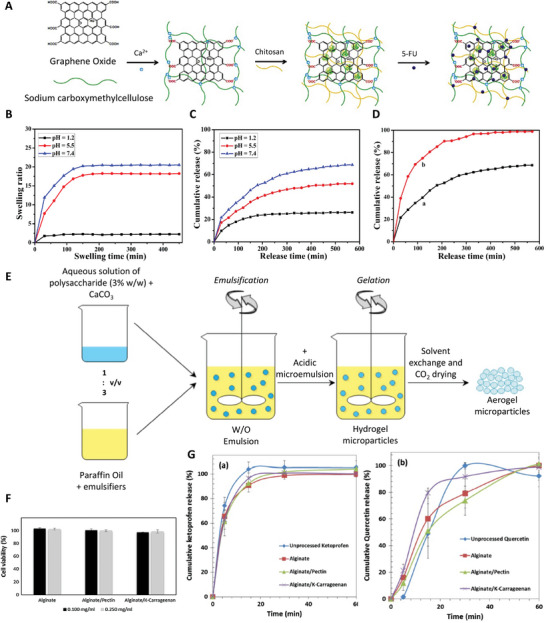
A) Illustration demonstrating the preparation steps of 5‐FU embedded hybrid aerogels. B) Changes in the swelling properties of the CS/CMC/Ca^2+^/GO hybrid aerogels with changing pH at a temperature of 37 °C. C) 5‐FU release from aerogels in 0.1 m PBS at specified pH at 37 °C. D) 5‐FU cumulative release from aerogels made of CS/CMC a) with Ca^2+^/GO and b) without Ca^2+^/GO immersed in 0.1 m PBS with pH 7.4 at 37 °C. E) Schematic showing the preparation of alginate‐based aerogel microparticles. F) MTS cytotoxicity testing: incubation of aerogels made of alginate (0.1 and 0.25 mg mL^−1^) in human colon carcinoma Caco‐2 cell line for 3 days. G) Release of ketoprofen (KET) (a), and quercetin microparticles. (b) Phosphate buffer solution (releasing media) with pH 6.8, at 37 °C. (A–D) Reproduced with permission.^[^ ^215^ ^]^ Copyright 2017, Elsevier. (E–G) Reproduced with permission.^[^ ^198^ ^]^ Copyright 2016, Elsevier.

In another study, hybrid aerogels were prepared from carboxymethyl cellulose (CMC), CS, and GO. Calcium ion (Ca^2+^) was used as the crosslinker.^[^ ^229^ ^]^ The pH‐controlled drug delivery performance of the resultant hybrid aerogels was investigated using a chemotherapeutic agent, 5‐FU. The process of drug loading on the hybrid aerogels is shown in **Figure** **15**A. The release of 5‐FU was observed to be pH‐sensitive due to the change in the aerogel swelling rate at various pH values. In addition, 5‐FU release from the prepared CS/CMC/Ca^2+^/GO–5‐FU aerogel was also expected to be pH‐responsive as both CMC and CS are pH‐sensitive polysaccharides. As shown in Figure 15B, the initial drug release was faster, which gradually slowed down for all tested pH values. The controlled release of 5‐FU at various pH values also indicated that the cumulative drug release from the hybrid aerogels peaked (release rate: 68%) when the pH value was 7.4 (Figure 15C). In addition, to investigate the effect of GO on the controlled release of 5‐FU, an additional experiment was conducted by loading 5‐FU into CS/CMC aerogel. As a result, the cumulative 5‐FU release via the CS/CMC carrier was consistently higher when compared to the release via the CS/CMC/Ca^2+^/GO carrier (Figure 15D). Overall, the results presented in this study offer potential for hybrid drug carriers derived from natural polysaccharides for use in controlled drug delivery applications.

The use of biopolymer hybrid aerogels as DDS has also received significant attention in the last decade because they are designed with relatively large surface area and easily accessible pore structures to enable high drug loading capabilities. For example, alginate‐based hybrid aerogel microparticles co‐gelled with methoxylpectin and *κ*‐carrageenan have recently been investigated as drug carriers delivered through mucosal tissue using model drugs, quercetin and KET.^[^ ^198^ ^]^ Alginate and alginate‐based hybrid microparticles were fabricated using emulsion gelation in conjunction with the internal curing technique (Figure 15E). The loading procedure, which involves emulsion gelation and CO_2_SCD, ensures that the antioxidant activity of quercetin is preserved and the hybrid microparticles form mesoporous structures with a significantly high specific surface area (>300 m^2^ g^−1^) and improved mucosal adhesion. Additional testing was performed to evaluate the biocompatibility of the hybrid aerogel microparticles. The results confirmed that the aerogel did not induce cytotoxic effects at the concentrations used even after 3 days of incubation (Figure 15F). Finally, the alginate/*κ*‐carrageenan aerogel matrix enabled slightly faster drug release for quercetin and KET than alginate/pectin counterparts (Figure 15G). Overall, the results of this study demonstrate the promising use of these hybrid microparticles as effective DDSs for mucosal applications.

A comprehensive overview of the application of aerogels in drug delivery has also been included (**Table** **6**). Overall, aerogel‐based systems appear to be a promising platform for use in drug delivery applications. The studies conducted so far demonstrate that aerogel‐based carriers have high drug loading capacities, controlled/sustained drug release ability, and increased bioavailability and stability of drugs with low solubility. The flexibility of the fabrication and surface functionalization steps can also modify the pore volume and surface area of the aerogel along with the surface chemistry, which in turn critically affects the drug adsorption and release rates. New approaches and materials, such as composite and layered aerogel matrices, pave the way for new possibilities to produce specific formulations for targeted applications. However, alternative functionalization and surface coatings are needed to facilitate the long‐term use of aerogels. Novel multi‐layered matrices should be designed to improve the drug delivery performance of currently used aerogel systems, thereby increasing the efficiency of controlled or sustained release of drugs. As such, the production of hybrid aerogels combining high surface area inorganic constituents with biodegradable organic components may enhance the performance of these promising materials, especially for drug delivery purposes, and introduce new materials with unique properties.

**Table 6:** A comprehensive overview of aerogel's performance in drug delivery application

Drug	Material	Drying	Aerogel Porosity %	Pore diameter size [nm]	Surface Area [m^2^ g^−1^]	Pore volume [cm^3^ g^−1^]	Drug loading [wt.%]	Amount of drug released [V%]	Release duration [h]	Ref
Ketoprofen (KET)	Alginate‐based gel particles	SCD	–	27	512 ± 2.15	–	18.05 – 28.95	100%	2	[208]
Nimesulide							5.50 – 14.96		4	
Loratadine							24.32 – 30.58		0.23	
Bendamustine Hydrochloride	Cellulose nanofiber	FD	–	–	31–33	–	0.81 ± 0.43 – 20.16 ± 2.64	69.205% ± 2.5% @ pH 1.2	24	[199]
								78% ± 2.28% @ pH 7.4		
Ketoprofen (KET)	Alginate; Alginate/pectin; Alginate/*κ*‐carrageenan	SCD	–	–	370–548	1.7 – 5.9	17–22	–	1	[198]
Quercetin							3.1‐ 5.4			
5‐fluorouracil	Chitosan, carboxymethyl cellulose, and graphene oxide aerogel using Ca^2+^ as the crosslinker	FD	–	–	–	–	54	26% @ pH 1.2; 51% @ pH 5.5; and 68% @ pH 7.4	9.67	[215]
Ketoprofen (KET)	Silica	SCD	> 85	14 – 18	1000 ± 50	–	15.38 ± 1.80	–	–	[46]
	Alginate				524 ± 26.4		11.83 ± 0.61			
	Pectin				397 ± 19.9		14.01 ± 3.84			
	Starch				127 ± 6.4		12.84 ± 0.92			
Benzoic acid	Silica				1000 ± 50		23.77 ± 0.12			
	Alginate				524 ± 26.4		18.92 ± 1.32			
	Pectin				397 ± 19.9		14.66 ± 1.24			
	Starch				127 ± 6.4		21.54 ± 2.04			
Ibuprofen (IBU)	*κ*‐carrageenan	SCD	–	8.5 – 13.5	60 – 174	0.17 – 0.37	35.00 ± 8.83 – 70.00 ± 6.68	99.4% – 100%	2	[202]
Silymarin	Gum tragacanth‐PVA	SCD	92.68± 0.21 – 93.98± 0.14		657.66 ± 2.47 – 1029.20 ± 20.19	19.34 – 25.28	29.82 ± 1.6 – 45.57 ± 2.3	–	–	[230]
Ibuprofen (IBU)	Silica‐gelatin	SCD	–	50–60	644	4.95	24	100% in 10 mm PBS pH 7.4	0.083	[205]
Ketoprofen (KET)							14	100% in HCl PH 2.0		
5‐fluorouracil	Carboxyl‐functionalized MWCNT with konjac glucomannan	FD	–	–	–	–	16.52 ± 0.45 – 32.25 ± 1.57	48% at pH 1.2 and 62% at pH 6.8	11	[216]
Ibuprofen (IBU)	Silica‐gelatin	SCD	–	17 – 32	285 ± 32 – 812 ± 61	1.69 – 6.20	19 – 24	–	–	[205]
Ketoprofen (KET)							11 – 15%			
Ibuprofen (IBU)	Starch	SCD	–	7.2 ± 0.01	72.5 ± 10.5	0.47 ± 0.07	29	80% at pH 7.4	1.5	[231]
Indomethacin	Alginate grafted with a copolymer of N‐isopropylacrylamide and N‐hydroxymethylacrylamide	FD	97.9	1000 – 5000	–	–	13.24	At 42 °C almost 100% at pH 7.4 and 10% at pH 2.1	26	[209]
Sodium salicylate	Polyethylenimine‐grafted cellulose nanofibrils	FD	–	–	79	–	78 mg g^−1^ at pH 3.0	64.31% at pH 2 and 92.59% at pH 7.4; 52.66% – 78.49% as temperature increases from 20 – 50 °C	24	[203]
5‐chloro‐2‐methyl‐4‐isothiazolin‐3‐one and 2‐methyl‐4‐isothiazolin‐3‐one	Fenugreek gum	FD	–	–	–	–	–	–	25	[225]
Methotrexate	Silica‐gelatin	SCD	–	25 – 30	544 ± 20	–	16	< 3% in buffer (pH 2.0, 5.0, and 7.4); < 3% in DMEM‐HAM'S F12; 8.6 ± 1.9% in SCC VII cell culture; 11.8 ± 2.0% in cell culture supernatant; 8.7 ± 1.2% in induced cell culture lysate; 11.6 ± 1.4% in induced cell culture supernatant; 4.6 ± 0.9% in cathepsin B in PBS	72	[217]
Ibuprofen (IBU)	Iro(III)‐crosslinked alginate	SCD	–	4 – 200	316 ± 19 – 442 ± 25	1.2 ± 0.2 – 1.8 ± 0.2	1.14 ± 0.02 – 1.8 ± 0.1	–	1.67	[200]
Resveratrol	Silica	FD	–	–	–	–	19	60% in the first 30 mins in both SGF (pH 2.0) and PBS (pH 7.4), with significantly slower release after	7	[232]
Gallic acid	Alginate	SCD	–	9 to 34	516 – 698	1.15 – 5.66	0.193 – 0.83 g g^−1^	–	–	[233]
Lysozyme	Fenugreek gum	FD	–	–	–	–	–	–	–	[226]
Proanthocyanidins	Alginate/pectin	FD	65.60 ± 0.05 – 79 ± 0.07	183 ± 9 – 1081 ± 10	–	–	9.62 ± 0.2 – 15.91 ± 0.11	–	4	[234]
Cisplatin	Chitosan‐Alginate	SCD	–	13.38	86	–	> 76%	60% in the first 2h	4	[235]
Doxorubicin	Graphene	–	–	755	–	–	253	60% in acidic acetate buffer (pH 5.4) and 38% in PBS (pH 7.4)	24	[220]
Vancomycin	Chitosan	SCD	96.8 – 97.2	12.6 – 15.0	257 – 479	1.01 – 1.70	8.5 ± 4.0 – 27.3 ± 2.8 ug mg^−1^	–	48	[222]
Ketoprofen (KET)	Silica	SCD	–	9.53 ± 0.8 – 24.44 ± 1.3	568.90 ± 26 – 895.40 ± 35	–	15.6 ± 1.5 – 23.5 ± 1.1	100% within first 50 min for lower density; 80% within first 50 min for higher density	3	[207]
Curcumin	Pectin	SCD	96.0 ± 0.05	–	441 ± 6	–	–	No release for the first two hours in SGF (pH 1.2), followed by the complete release in an hour in SIF (pH 6.8)	24	[212]
	Chitosan‐coated pectin		94.8 ± 0.03		276 ± 8			No release for the first two hours in SGF (pH 1.2), followed by a slower release for up to 24 h		
Camptothecin	KCC‐1 nanofibrous silica microparticles functionalized with methyl groups, PVA, and PAA hybrid	FD	–	–	–	–	97.0 ± 0.6 – 97.7 ± 0.1	–	30 – 38 days	[218]
Doxorubicin	PEI‐grafted cellulose nanofibers	FD	–	–	–	–	330.12 mg g^−1^	59.45% at pH 3 and 37 °C	16	[214]
Polymyxin B, Nisin, and lysozyme	Fenugreek gum	FD	99.91	9830	11.23	–	–	–	–	[226]
	Sesbania gum		96.19	6940	16.89					
	Guar gum		93.28	7680	15.54					
Ibuprofen (IBU)	Carbon	FD	–	7.9 – 12.7	314 – 343	.61 – 1.03	18.2 ± 0.77 – 22.9 ± 1.05	At pH 6.5, 14 – 45% released in the first 5 mins; At pH 5.0, 8 – 23% released	2	[201]
Genistein	Starch	FD	–	3.85 ± 0.16	6.6 ±1.2	0.0974 ± 0.022	4.24 ± 0.04 – 49.01 ± 0.66	65% and 60% released within 6 h in pH 6.8 and 1.2 respectively	24	[221]
Curcumin	Starch	SCD	92.8 ± 0.2	19.9 ± 2.5	60.4 ± 3.0	0.26 ± 0.01	224.2 mg g^−1^	–	–	[211]
Ketoprofen (KET)	Anionic nanofibrillar cellulose	FD	–	–	–	–	3.4	–	144	[213]
Nadolol							1.7	–		
Metronidazole							2	–		
FITC‐dextran							1	85 – 100%		
Lysozyme							0.5	20 – 30%		
Bovine Serum Albumin							1	≈40%		
Diclofenac sodium	High methoxyl pectin and xanthan	SCD	–	6.8	289	0.11	4.5 ± 0.2	100%	24	[210]
Indomethacin							4.2 ± 0.3			
Amoxicillin	Cellulose	FD	–	–	–	–	4.03 ± 0.07 – 13.45 ± 0 ug cm^−2^	–	12	[224]
Doxycycline	Alginate outer layer and pectin core	SCD	–	–	–	–	–	–	32 – 54	[223]
Salbutamol	Chitosan	SCD	–	–	–	–	–	–	–	[202]
		FD								
Dexamethasone	Mesoporous silica NPs, PVA, and PAA	FD	–	–	252 – 303	–	5.1 ± 0.1 – 6.0 ± 1.6 mg g^−1^	30 – 70%	40 – 90 days	[227]

### 5.3 Tissue Engineering

Tissue engineering is a field of research that develops biological structures that improve or restore tissue function using various fields such as life sciences and engineering.^[^ ^49^, ^236^ ^]^ To engineer specific tissues, cells are surrounded by a supporting structure that recapitulates the 3D configuration of the native tissue. 3D scaffolds can create such structures by mimicking the particular mechanical, physical, or biological properties of the native target tissue.^[^ ^49^ ^]^ Aerogels, highly porous biomaterials fabricated from a variety of macromolecules, composites, and inorganic materials, recapitulate the construction of the ECM, a 3D network of macromolecules such as glycoproteins and collagen (COL). Aerogels can provide structural support and demonstrate biochemical properties to cells within tissues.^[^ ^35^, ^237^ ^]^

A key characteristic of aerogels for tissue engineering and regenerative medicine is their 3D nanostructured mesoporosity for cell adhesion, ensuring interconnectivity to supply cells with oxygen and nutrients and remove metabolic wastes.^[^ ^238^ ^]^ These biomaterials can be fabricated into a variety of shapes required for scaffolds.^[^ ^191^ ^]^ However, aerogels generally lack the macroporosity required to promote tissue growth and bone angiogenesis and ingrowth. Besides, bone tissue scaffolds generally require high mechanical stress, whereas conventional aerogels have low mechanical properties. Therefore, to overcome these limitations, research on aerogels for bone tissue engineering applications is mostly focused on designing advanced aerogels by exploring ways to enhance their mechanical properties, biofunctionalization, and macroporous structure. To improve the mechanical properties of aerogels, various mixtures such as inorganic fillers or biopolymers have been presented to improve the stiffness and promote ductile deformation.^[^ ^239^ ^]^ This strengthening of aerogels can be accomplished by immersing the wet gel in a solution containing the strengthening agent.^[^ ^240^ ^]^ The strengthening biopolymer can be precipitated into a gel by an antisolvent. The next step is to proceed with the drying process by SCD of the gel or to use a one‐step CO_2_SCD‐assisted anti‐solvent precipitation/drying method. Through this method, various biopolymer distributions can be achieved on the aerogel scaffold.^[^ ^191^ ^]^ The resulting scaffolds strengthened with low amounts of biopolymers are shown to support cell proliferation and adhesion.^[^ ^241^ ^]^

Various approaches can be used to generate macroporosity in mesoporous aerogels,^[^ ^241^ ^]^ resulting in scaffolds with dual‐porosity (i.e., meso and macro). Porogens (i.e., paraffin, PMMA, NaCl, D‐fructose) of controlled shape and size can be exploited as sacrificial prototypes to provide microporosity to the aerogels.^[^ ^241^ ^]^ Another approach to creating macroporosity is to use emulsion templates. The continuous phase is gelled, and the dispersed phase is removed either by SCD^[^ ^238^ ^]^ or by rapid pressure relief after SCD.^[^ ^88^ ^]^

In the following sections, we review some of the applications of aerogel‐based biomaterials in bone, skin, and nerve tissue engineering.

#### 5.3.1 Bone

Bone constitutes the skeleton of vertebrates, serves to protect and support the body, and is a major site for the production of red and white blood cells and the accumulation of minerals. Due to its intricate external and internal structures, it is rigid and strong. Typically, on a dry weight basis, bone consists of about 70% minerals (hydroxyapatite (HA) nanocrystals) and about 30% organic matter (glycoprotein and COL).^[^ ^236^ ^]^

**Figure 16:**
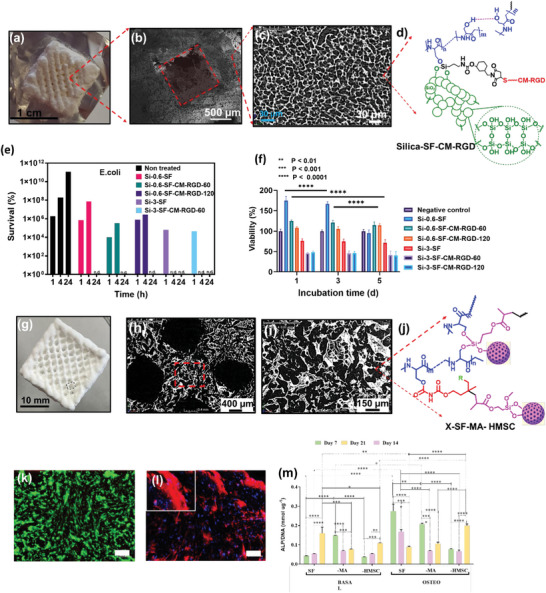
3D‐printed Si‐based aerogel construct (Si‐3‐SF‐CM‐RGD‐60): a) Optical image, b) SEM image, c) X‐ray scan, d) the molecular structure, e) antimicrobial activity against 1 × 10^6^ E. coli over time, and f) the viability test toward MC3T3‐E1 osteoblastic cell line. Adapted with permission.^[^ ^11^ ^]^ Copyright 2021, American Chemical Society. 3D printed SF‐MA‐30‐HMSC‐2 aerogel construct: g) Optical image, h,i) SEM image, and j) X‐ray scan of the porous structure. k,l) Fluorescence images (hMSC after 21 d in culture) under BASAL conditions. (k) Stained liver cells by calcein‐AM (green) and dead cells by propidium iodide (red). Scale bars correspond to 200 µm. (l) Stained DNA by DAPI (blue), and F‐actin filaments stained by Phalloidin (red). Scale bars correspond to 100 µm. m) ALP activity under BASAL and OSTEO conditions (21 days). The ALP values were normalized by DNA content. *p<0.05; **p<0.01; ***p<0.001; ****p<0.0001. Reproduced with permission.^[^ ^181^ ^]^ Copyright 2022, John Wiley and Sons.

A combination of 3D printing and directional freeze casting of the wet gel can equip the aerogels with an open porous structure necessary for osteoblast ingrowth and proliferation. Recently, Maleki et al. developed antibacterial and osteoconductive biomimetic hybrid aerogels‐based scaffolds through a synergetic combination of sol‐gel reaction, microextrusion‐based 3D printing, and directional freeze casting of silica/hollow mesoporous silica capsules (HMSC)‐SF composite aerogels with multi‐scale porosities.^[^ ^11^, ^181^, ^242^ ^]^ The scaffolds demonstrated a combination of improved compressive behavior and excellent printability controlled by tuning the sol‐gel parameters of organosilanes and/or HMSC content, self‐assembly in SF and methacrylated SF (SF‐MA) biopolymer.^[^ ^11^ ^]^ While nanosized pores in final aerogels facilitate protein absorption and nutrient and oxygen delivery to the osteoblast, the macro‐sized pores developed by printing ensure osteoblast infiltration and growth inside the aerogel network.^[^ ^181^ ^]^ As shown in **Figure** **16**, the pore interconnectivity developed by freeze‐casting facilitated the final aerogel scaffold with pore interconnectivity in the printed struts. The same group has also demonstrated that the bio‐conjugation of (Arg)−glycine (Gly)−aspartic acid (Asp) (RGD)‐AMP on the SF through maleimide coupling reaction prior to the self‐assembly/gelation confer an antibacterial activity toward various bacterial strands with enhanced cellular attachment on the scaffold surface (See Figure 16(e,f)). In addition, embedding the ciprofloxacin‐loaded methacrylate HMSC (HMSC‐MA) to the SF‐MA and subsequent simultaneous 3D printing and photocrosslinking of the resultant gels led to obtaining mechanically stable constructs with improved antibacterial, human mesenchymal stem cells colonizations, and differentiation as it is evident from Figure 16(j–m).^[^ ^181^ ^]^

**Figure 17:**
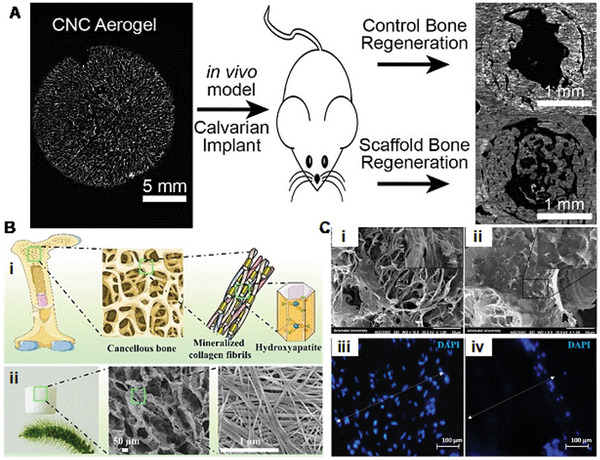
Microstructure of aerogel‐based biomaterials in tissue engineering: A) Crosslinked cellulose nanocrystal (CNC) aerogels. Reproduced with permission.^[^ ^243^ ^]^ Copyright 2020, Elsevier. B) i) Representation of the microstructure of the cancellous bone. ii) SEM images of the HA nanowire aerogel. Reproduced with permission.^[^ ^248^ ^]^ Copyright 2018, American Chemical Society. C) Cells’ morphology on the scaffolds; SEM: i) the fibroblast cells, and ii) keratinocytes on the N1 and N2 layers, respectively. DAPI staining of the: iii) fibroblast‐seeded N1 and iv) keratinocyte–seeded N2 layers; arrows displayed the size of (iii) N1 and (iv) N2 layers. Reproduced with permission.^[^ ^49^ ^]^ Copyright 2020, Elsevier.

Chemically crosslinked cellulose nanocrystal (CNC) aerogels have various characteristics that make them useful for bone tissue engineering applications. Hydrazone crosslinked aerogels were generated using various types of CNC (sulfated or phosphate) and then assessed in vitro using osteoblast‐like cells (Saos‐2 cells).^[^ ^243^ ^]^ They were found to have enhanced metabolic activity of the cells for more than 7 days, whereas alkaline phosphatase analyses indicated that the cells preserved the phenotype. All fabricated aerogels showed HA growth for up to 2 weeks during immersion in simulated body fluid (SBF) solution and pre‐treatment with 0.1 m CaCl_2_ solution. The sulfated CNC aerogels moderately outperformed the phosphate CNC aerogels for compressive strength and continuous stability in liquids. To verify the effect of aerogels, modified CNC aerogels were implanted in the calvarial bone defect model of fully‐grown Long Evans rats. Compared to control samples at 3 weeks and 12 weeks, increased bone volume by 33% and 50%, respectively, had been observed using sulfated CNC aerogels. Their findings show that crosslinked CNC aerogels are porous, flexible, and successfully allow bone growth after embedding in the bone defects (**Figure** **17**A).^[^ ^243^ ^]^

In another study, Reyes‐Pieces et al. reported a novel synthetic method to attain a CS‐silica hybrid aerogel with a CS content of about 10 wt.% using 3‐glycidoxypropyl trimethoxysilane (GPTMS) as a coupling agent.^[^ ^244^ ^]^ Aerogels were made via the sol‐gel method and CO_2_SCD, resulting in aerogels with densities ranging from 0.17–0.38 g cm^−3^. Textural assays by N_2_‐physical absorption revealed a highly interconnected network of mesopores with a reduced specific surface area (1230–700 m^2^ g^−1^) and pore sizes ranging from 11.1–8.7 nm. In addition, samples showed very rapid swelling through spontaneous capillary inhibition in Phosphate‐buffered saline (PBS). The generation of covalently crosslinked hybrid structures was proposed by Fourier‐transform infrared spectroscopy (FTIR) and validated with over 400‐fold improvement in compressive strength. The aerogel exhibited bioactivity in SFB, as confirmed by the in vitro generation of an HA layer with a crystal size of around 2 µm. The cell study indicated a mechanosensitive response and a non‐cytotoxic effect on HOB osteoblasts.^[^ ^244^ ^]^

Recently, aerogels composed of polybenzoxazine (PBO) have also been employed for bone tissue engineering. PBO is produced from aniline and bisphenol A, precursors to various frequently applicable materials such as PU. Specifically, this novel aerogel mimics the native ECM of bone tissue. If these aerogels show desirable biocompatibility, they can be employed as scaffolds for bone tissue engineering. Rubenstein et al. reported that carbonized PBO‐based aerogels offer a promising approach to bone tissue engineering due to improved ALP activity and osteocalcin accumulation when compared with other PBO aerogel formulations.^[^ ^238^ ^]^ To do this, they tried to determine some physical properties of these aerogels. They evaluated biocompatibility for human osteoblasts using PBO‐based aerogels co‐polymerized with formaldehyde (RF) and resorcinol and converted to a carbon aerogel. In addition, metabolic activity, osteocalcin production, and alkaline phosphatase activity were investigated after culture for up to 5 days. Co‐polymerization of RF and PBO‐based aerogels showed a low value in porosity, density, and elastic modulus. In terms of bone cell growth, these aerogels as substrates showed the weakest performance. On the other hand, PBO‐based carbon aerogels have high porosity, high density, and enhanced mechanical properties and provide the most desirable substrate for bone cell growth. These findings suggest that PBO‐derived carbon aerogels offer adequate biocompatibility for osteoblasts and can be used for bone tissue engineering scaffolds.

Electrospun nanofiber‐reinforced aerogels can also be applied to bone tissue engineering. Zhang et al. produced a combination of CS aerogels with cellulose acetate (CA) and poly (ɛ‐caprolactone) (PCL) nanofibers through ball milling and freeze‐drying methods.^[^ ^245^ ^]^ In their study, SEM analysis, FTIR spectrum, X‐ray photoelectron spectroscopy (XPS), compressive experiment, and in vitro cell studies were performed to evaluate physicochemical properties and biological performance. The SEM analysis revealed that the desired morphology was achieved in the CS/PCL/CS aerogels with the combination of CA/PCL nanofibers and CS solution. The FTIR and XPS examinations showed high integration of CA, PCL, and CS. Compressive testing confirmed that CA/PCL/CS aerogel improved the compressive modulus of pure CS aerogel. In vitro studies further confirmed that the CA/PCL/CS composite scaffolds exhibited better cytocompatibility compared to pure CS. In addition, cells cultured in CA/PCL/CS showed well‐expanded morphology and were able to penetrate into porous scaffolds. Moreover, confocal testing showed that CA/PCL/CS could promote osteogenic differentiation of MC3T3‐E1 cells. In summary, CA/PCL nanofibers not only enhanced the mechanical characteristics of CS‐based aerogels but also improved osteogenic differentiation and cell attachment.^[^ ^245^ ^]^

A combination of aerogels with carbon‐based nanomaterials has been presented for bone tissue engineering. Muñoz‐Ruíz et al. designed a highly porous aerogel‐based scaffold on COL‐alginate‐GO.^[^ ^237^ ^]^ GO production was performed using the “Hummer method”. COL‐alginate and COL‐alginate‐GO hydrogels were synthesized and then exposed to SCD. The produced aerogels were investigated by FTIR and SEM. Osteoblasts were cultured on these scaffolds and examined using SEM. The aerogels showed a densely interconnected network with a non‐porous external wall. FTIR analysis revealed that the functional groups of GO and COL were preserved following the SCD procedure. SEM results further showed that a scaffold composed of COL‐alginate promotes cell proliferation and adhesion.^[^ ^237^ ^]^

Conductive carbon‐based scaffolds have received considerable interest as they show that electrical stimulation can significantly influence cell behavior. However, the high‐temperature requirement for the synthesis of aerogels negatively affects the stability of the polymer network. Tevlek et al. reported the synthesis of a carbon‐aerogel scaffold combined with ceramic NPs of tricalcium phosphate.^[^ ^246^ ^]^ The scaffolds can be made at high temperatures (≈ 1100 °C) without substantial impact on the morphology of the composite, resulting in a structure similar to the original aerogel. Although the conductivity of the composite material can be reduced by enhancing the ceramic content, the observed conductivity values were close to those previously reported for polymer‐functionalized carbon aerogels. Cell experiments have shown that the designed scaffolds promote cell adhesion and proliferation, suggesting potential scaffolds for tissue engineering applications. In summary, porous, conductive, biocompatible, and non‐toxic scaffolds fabricated from carbon‐based aerogels containing ceramic nanocrystallite integrated fibers can be employed for tissue engineering applications that require porous conductive materials.^[^ ^246^ ^]^

Aerogels produced from natural polymers have been studied extensively for bone grafting due to their high biocompatibility and high porosity. However, the mechanical strength of aerogels made of natural polymers is very low to treat large bone defects. GO is one of the most suitable nanomaterials with desired mechanical characteristics and biocompatibility and is a promising factor for the fabrication of hybrid aerogels. Liu et al. developed an aerogel composed of GO and type I COL with a highly porous structure using the sol‐gel method for various concentrations of GO.^[^ ^247^ ^]^ Their results showed that the GO–COL aerogels are hydrophilic and densely porous. Moreover, the compressive strength of GO–COL aerogels was improved by enhancing the GO concentration. In vitro experiments revealed that 0.1% GO–COL aerogel showed improved cytocompatibility and biomineralization compared to other aerogel groups. Additionally, the in vivo experiments showed that the 0.1% (w/v) GO–COL aerogels exhibited superior bone repair performance compared to COL aerogel in a rat cranial defect model. These investigations demonstrated that the 0.1% GO–COL aerogel provided the better osteogenic ability and in vivo biocompatibility, suggesting it is an advantageous material for bone tissue engineering.^[^ ^247^ ^]^

To recapitulate the structure of cancellous bone, Zhang et al. used a novel approach to fabricate HA nanowire aerogel scaffolds with a low density (8.54 mg cm^−3^), porous meshwork, and 3D interconnected microstructure.^[^ ^248^ ^]^ The HA nanowire aerogels showed high elasticity and high porosity of ≈99.7%, very similar to the properties of the porous network of cancellous bones (Figure 17B).

#### 5.3.2 Skin

Skin, the largest tissue in the body, is composed of two layers, the epidermis and dermis. The dermis is the lower layer, which is thicker than the other layers and is more porous and flexible. The dermis consists mainly of connective tissue and nourishes the epidermis layer. The epidermis represents the upper layer of the skin and is composed of keratinocytes with low porosity and squamous structure, forming a protective layer against pathogenic microorganisms.^[^ ^49^ ^]^

Polysaccharides and proteins are low‐cost, biocompatible, sustainable, and biodegradable, with structures similar to the ECM. Therefore, cellulose‐based aerogels with highly interconnected pores are promising biomaterial candidates for skin tissue engineering. These 3D scaffolds can be produced by environmentally friendly, scalable, and cost‐effective freeze‐drying methods. The mechanical, biological, and physicochemical properties of cellulose can be improved with the combination of proteins and other polysaccharides.^[^ ^249^ ^]^ Ghafari et al. fabricated an innovative bilayer scaffold composed of CNF/PVA and explored possible applications in skin tissue. They employed a single‐step freeze‐drying method at various concentrations of the mentioned polymers. Field emission‐SEM results showed that the created scaffolds comprised highly interconnected pores with two specific pore sizes in different layers of the scaffolds that can mimic epidermis and dermis layers. A lower concentration of polymers leads to larger pore size, increased porosity, improved water absorption, and decreased mechanical strength. FTIR analysis reveals strong hydrogen bonding between CNF/PVA molecules, as well as the presence of functional chemical groups and effective crosslinking. The cytotoxicity assessment via (3‐(4,5‐Dimethylthiazol‐2‐yl)‐2,5‐Diphenyltetrazolium Bromide) (MTT) assay demonstrated that the fabricated scaffolds provide biocompatible material for skin tissue regeneration (Figure 17C).^[^ ^49^ ^]^

In another study focused on applying aerogel‐based materials for skin tissue engineering, Piątkowski et al. designed a novel CS‐based aerogel with improved post‐burn wound healing properties.^[^ ^178^ ^]^ CS scaffolds containing gold NPs were achieved in microwave‐assisted conditions by eco‐friendly and biocompatible reagents. The chemical structure, biodegradability, morphology, cytotoxicity, and antibacterial activity of the prepared material were investigated. Their findings show that these biomaterials are bioactive and antibacterial, making them a promising biomaterial for regenerative medicine.

SF is also a type of aerogel derived from the *Bombyx mori* silkworm. SF‐based porous biomaterials are widely studied for a myriad of biomedical applications because of their biodegradability and biocompatibility. Mallepally et al. utilized CO_2_‐assisted acidification to produce SF‐based hydrogels and then convert them to SF aerogels.^[^ ^250^ ^]^ The SF concentration in the aqueous media is exploited to adjust the textural properties and morphology of the SF aerogels. The surface area of the SF aerogels after CO_2_SCD was about 5 times higher than that of SF cryogels. The SF‐based aerogels showed a unique pore morphology compared to the SF cryogels. Additionally, cell experiments on human skin fibroblasts exhibited the cytocompatibility of the SF‐based aerogel scaffolds and the presence of embedded cells in the aerogel scaffolds.

#### 5.3.3 Nerve Tissue

Currently, there is a lack of complete treatment solutions for central nervous system injury. Researchers set out to investigate innovative approaches that promote neural tissue regeneration while eliminating inhibitory fibroglial scars. For this purpose, Zeinali et al. studied the fabrication and characterization of a hybrid scaffold composed of GO aerogel/gelatin for nerve tissue engineering.^[^ ^10^ ^]^ In their study, a hollow GO aerogel was first produced. After that, a porous gelatin structure was created through thermal phase separation, and the gelatin structure was embedded into the aerogel. The mechanical‐thermal properties of the fabricated scaffolds were evaluated by Dynamic Mechanical Thermal Analysis (DMTA). At a temperature of −70 °C, the mechanical strength of the scaffold model and the fabricated hybrid scaffold were about 1.3 and 22.6 MPa, respectively. A substantial improvement was observed in the elastic modulus of the hybrid scaffold, demonstrating the strengthening of the aerogel in the fabricated scaffold. As observed in the cell experiments, the metabolic activity was significantly improved compared to the control sample. This high biocompatibility allows P_19_ cells to attach to and differentiate on the hybrid scaffold surface. Differentiation was close to 20% and 87%, respectively, on the control and scaffold surfaces, and the P_19_ cells successfully differentiated into neural cells. These findings showed that these aerogels are appropriate scaffolds for neural tissue engineering.^[^ ^10^ ^]^

In summary, natural polymer‐based aerogels are desirable for tissue engineering applications because they are generally biodegradable, have a structure similar to constituents of the tissue ECM, and exhibit favorable cytocompatibility.^[^ ^1^, ^251^ ^]^ It has been proposed to utilize mixtures such as lignin or bioceramics with aerogels to confer improved mechanical characteristics, osteoconductivity, and enhanced cell attachment to the resulting scaffolds.^[^ ^251^, ^252^ ^]^

The complexity of tissue engineering lies in the interaction of scaffolds, cells, nutrients, and signaling factors. Appropriate scaffolds can support cells and provide direction to create tissue. Further improvements to create 3D scaffolds are desired to construct larger‐scale (macro‐sized) tissue constructs with long‐lasting safety and adaptability. The development of scaffolds with interconnected pores is crucial because they recapitulate the functions of the ECM, and exploiting innovative 3D printing methods can provide the capability to adjust the physicochemical characteristics of such structures. In addition, surface modifications can be investigated by utilizing growth factors, peptides, and conductive polymers to further improve the scaffolds' function and promote cell proliferation and growth.^[^ ^249^ ^]^

### 5.4 Diagnostics

Electrophysiological monitoring for next‐generation sensing and diagnostics systems relies on the advancement of adaptive, safe, and smart materials with unique and superior electrical, mechanical, and biomedical properties. The use of such sensing systems, which allows for long‐term remote monitoring by providing high sensitivity and preferably low detection limits, has great potential in the healthcare field.^[^ ^253^ ^]^ The interface between the human body and the monitoring system plays a critical role in the diagnosis process, mostly accomplished by high‐performance electrodes.^[^ ^254^ ^]^ In the past decades, a myriad of electrodes based on advanced materials have been proposed^[^ ^253^ ^]^ and utilized for various applications such as biochemical sensors,^[^ ^255^ ^]^ electrocardiography (ECG),^[^ ^256^ ^]^ electromyography (EMG),^[^ ^257^ ^]^ and functional electrical stimulation.^[^ ^258^ ^]^ It is very important to develop electrodes that can function efficiently when used on the human body by preserving their mechanical and electrical properties. To fulfill these requirements, a recent study proposed a biocompatible electrode using a hydrophilic polymer, that is, cellulose, comprising MWCNTs as filler with conductive properties.^[^ ^253^ ^]^ The cellulose in nanocrystal form was used to construct the main structure of the aerogel film, and relatively long cellulose fibers were utilized to increase the flexibility of the film. The results indicated that the resulting aerogel exhibits an electrical impedance that can be effectively used as a wet electrode with high mechanical flexibility and exceptional water absorption for electrophysiological monitoring.

**Figure 18:**
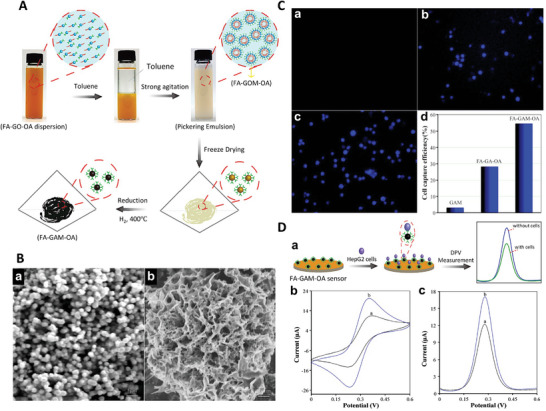
A) Synthesis of FA‐GAM‐OA aerogel. B) SEM images (a, b). C) Fluorescence microscopy images of the GAM (a), FA‐GA‐OA (b), and FA‐GAM‐OA (c) samples, and cell capture efficiencies (d). D) The sensing mechanism for detection of HepG2 cells via the FA‐GAM‐OA sensor (a), the CV curves (b), and DPV curves (c) of FA‐GA‐OA sensor in a 5 mm K_4_Fe(CN)_6_ at the scan rate of 100 mV s^−1^ with (a) or without HepG2 cells. Reproduced with permission.^[^ ^260^ ^]^ Copyright 2018, Elsevier.

Another important application of functional aerogel materials is for cancer diagnosis. Early diagnosis and treatment of cancers are crucial to prevent metastasis. As a result, diagnostic methods utilizing aerogels have started to gain significant attention due to their unique properties, enhanced conductivity and electronic interactions, electrochemical properties, mechanical strength, and high surface area.^[^ ^259^ ^]^ A recent study reported an electrochemical sensor developed for the detection of cancer cells. The sensor was composed of folic acid (FA) and octadecylamine (OA)‐functionalized GA aerogel microspheres (called FA‐GAM‐OA) (**Figure** **18**).^[^ ^260^ ^]^ The FA‐GAM‐OA aerogel synthesis steps include the Pickering emulsion preparation, FA‐GO microsphere (GOM)‐OA, and FA‐GAM‐OA (Figure 18A). The developed FA‐GAM‐OA exhibited a spherical morphology with a diameter of ≈1.2 µm, composed of open pores and FA groups (Figure 18B). The FA‐GAM‐OA aerogel sensor was employed to detect liver tumor cells (HepG2) in blood and showed a sensitive and selective electrochemical response with high capture efficiency (Figure 18C). This suggested method has the advantage of easy sensor preparation. In addition, it showed excellent performance in terms of detection sensitivity and selectivity of liver cancer cells (Figure 18D), demonstrating its successful use in early diagnostics and cancer detection.

Aerogel‐based diagnosis methods have also attracted many researchers to detect blood sugar levels in real‐time as an accurate reading for the prevention or treatment of diabetes. In a recent study, Xu et al. demonstrated an enzymatic microfluidic biosensor made of a 3D porous GA and glucose oxidase (GOx) for electrochemical glucose detection.^[^ ^261^ ^]^ The GA enabled high electrical conductivity, and the 3D porous structure supported the necessary biological conditions and was able to immobilize more GOx on GA due to the increased specific surface area. The biosensor was observed to provide good sensitivity, selectivity, and stability due to various integrated properties such as conservation of GOx by the 3D structure of the aerogel, the specificity of GOx, and low level of detectability. Furthermore, the microfluidic biosensor was tested for glucose detection and detected various glucose concentration levels in human serum samples relevant to practical situations, further validating the feasibility of the proposed detection method.

Recently, nanocellulosic aerogel (NA) has also been used in biosensor design and diagnostic applications. This is because NA provides a lightweight material with favorable structural and mechanical properties such as interconnected dense pores and high specific surface area, as well as biocompatible properties. Edwards et al. studied the synthesis and performance of peptide‐nanocellulose aerogels (PepNA) produced from raw cotton for protease detection.^[^ ^262^ ^]^ Greige cotton was used to prepare low‐density cellulosic aerogels by dissolving cellulose using calcium thiocyanate octahydrate/lithium chloride medium. The resulting cotton‐based aerogel had 99% porosity with mesopores with a size of 2–50 nm. The bioactivity of the prepared PepNA was observed to be high, and the detection sensitivity for an inflammatory diagnostic biomarker, that is, human neutrophil elastase, was measured to be 0.13 U mL^−1^. Overall, the study results showed that the physical and biochemical properties of the developed aerogel were sufficient for successful integration with protease‐sequestrant wound dressings.

**Figure 19:**
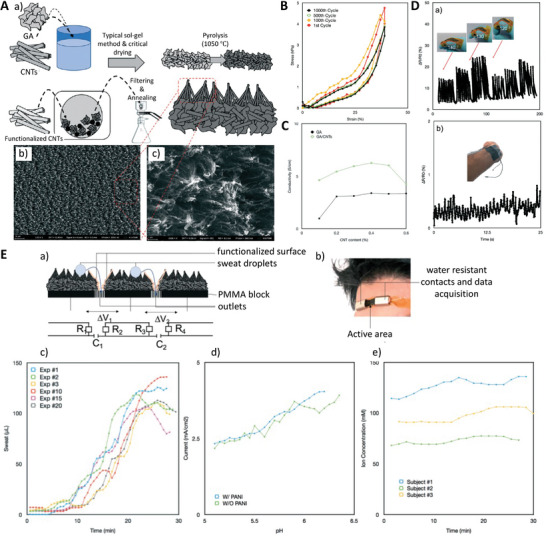
A) GA/CNT: a) synthesis schematic. b) SEM image of GA/CNT and c) SEM image of CNT structure. B) Cycling performance of GA/CNTs. C) Electrical performance of GA/CNTs. D) a) Strain sensing with different angles of the finger. b) The real‐time monitoring of pulse. E) a) GA/CNTs diagram, b) AB‐SSS on the human forehead, c) study of the sweat collection measurement during exercise, d) current against pH‐level performance of sample with and without PANI change, and e) ion concentration study during exercise. Reproduced with permission.^[^ ^263^ ^]^ Copyright 2018, Royal Society of Chemistry.

The application of novel aerogels has also been extended to the chemical and mechanical monitoring of human movement. A recent study demonstrated a novel aerogel‐based sensor that effectively extracts information via microfluidic sweat sampling originating from involuntary physiological human motions. GAs was supported by CNTs to construct highly stretchable 3D hybrid structures, thus preserving the function of conductive channels even at high levels of strain or stress (**Figure** **19**).^[^ ^263^ ^]^ PDMS@silk (1% silk) complexes were also ingrained in GA/CNTs to enact desired piezoelectric properties, allowing direct contact with the skin while displaying improved stretchability and sensitivity. The fabrication of the strain sensor is shown in Figure 19A, illustrating the conformal coating produced by CNTs on the surface of the aerogel having a pyramid form. The proposed aerogel‐based strain and sweat sensor (AB‐SSS) demonstrated exceptional robustness (Figure 19B), electrical conductivity (Figure 19C), and stretchability (Figure 19D). A microfluidic device was fabricated after confirming successful device performance by forming tiny microfluidic gaps in the GA/CNT framework. A microfluidic gap was prepared by obstructing the pores of the filter paper during the filtering process. The sensor outputs were observed to be stable during the experiments with good reproducibility (Figure 19E). Overall, the findings of this study can pave the way for developing personalized preventive healthcare systems for the correlation of multiple biometric information in real‐time.

The unique properties of aerogel formulations such as tunable composition, high porous network, and high mechanical strength have revealed potential in many tissue engineering applications. Aerogels with customizable properties and superior durability have been found to be particularly applicable to biosensors, non‐invasive imaging, and diagnosis devices. Specifically, the porous structure and large surface area of aerogels have contributed to their utilization as matrices for biomolecule detection and sensing applications. Therefore, it is believed that aerogels will rapidly evolve in the biomaterials market, expanding their use in biomedical applications with new aerogel formulations with improved mechanical properties and long‐term sustainability while maintaining their inherent characteristics.

### 5.5 Pressure Sensing

With the fast growth of artificial intelligence, novel materials for biomimetic robot technology as well as smart sensing, are required.^[^ ^264^ ^]^ A pressure sensor with the ability to transduce strain into resistance and capacitor has drawn major attention to emerging wearable functional electronics.^[^ ^265^ ^]^ Most of the reported aerogel and aerogel‐like pressure sensors functioned based on a piezoresistive pressure sensing mechanism. For this, creating an electrical conduction path in the porous network of aerogel and the mechanical resilience of the network are required to function optimally in a pressure‐sensing device. Considering these criteria, most of the aerogels reported so far have been fabricated from electrically conducting building blocks, usually 1D and 2D carbon allotropes^[^ ^266^ ^]^ and ceramics.^[^ ^267^ ^]^ Several piezoresistive sensors made of MXene/reduced GO, polyimide/rGO aerogel,^[^ ^268^ ^]^ and nacre‐mimetic rGO/PVA film^[^ ^269^ ^]^ have been studied, all of which have presented great potential in the transduction of cyclic compression to resistance changes even at varied pressure ranges.^[^ ^270^ ^]^

The super‐flexible aerogels based on aminosilane‐crosslinked (AC) reduced GO (AC‐rGO) aerogels were prepared via simple crosslinking of GO nanosheets using various kinds of aminosilanes. It is found that 3‐ aminopropyl(diethoxy)methyl silane (APDEMS) is the more promising choice to improve the hydrophobicity and elasticity of the resulting aerogels. Among variously studied aminosilane, APDEMS molecule was the optimal choice as it could act simultaneously as a crosslinker reductant and hydrophobizing agent of GO. The AC‐rGO were folded with a high surface area, ultralow density, super‐hydrophobicity, and super compressibility, making them optimal materials for strain and pressure sensing applications. The designed strain/pressure sensing arrays from AC‐rGO aerogels indicated an effective strain detection (0−80%) at relatively low pressure (10 Pa−10 kPa) distributions.^[^ ^271^ ^]^ Recently, aerogel‐inspired composites were developed by cryo‐assembly of SF biopolymer with MXene and GO nanosheets by Maleki et al.^[^ ^272^ ^]^ The porous architecture of composite aerogels was hierarchically assembled into the lamellae and interconnected bridges by inspiring from the nacre microstructure using the multipurpose freeze‐casting approach. The developed composite aerogel‐inspired structure exhibited a tunable porous structure composed of crosslinked SF chains entangled with MXene and GO nanosheets (X‐SF‐MXene). Such a structural complexity conferred great resilience to the final aerogels, which was not straightforward to achieve by conventional fabrication techniques such as sol‐gel approaches. The X‐SF‐MXene aerogels combined a low density (6−60 mg cm^−3^), compressibility (up to 30% without failure), and electrical conductivity rendering them the great pressure‐sensing capability for the detection of strain (up to 50%) and pressure (≈1 Pa to 2 kPa) distributions.

### 5.6 Other Potential Applications

Aerogels can be potentially used for gene delivery applications. By concept, genes can be embedded in the aerogel structure during gelation and be released in a particular location through either release‐by‐degradation or release‐by‐swelling of the aerogel subjected to liquid absorption/adsorption. Another interesting application for aerogels in the biomedical world could be medical imaging. The investigation of the ultrasonic properties of highly interconnected aerogel‐based implants in numerous insertion depths of the human cadaver model showed robust contrast to nearby tissue with isoechoic behavior.^[^ ^273^ ^]^ It is believed that the porous structure of aerogel can act as a multipurpose carrier for the encapsulation of La_2_O_2_S:Eu (0 to 50%) as the application of thermographic X‐ray phosphor dopant.^[^ ^274^ ^]^

## 6 Conclusion and Outlook

In this review, the synthesis and emerging aerogel processing technologies with a focus on their various biomedical applications are reviewed. As revealed here, aerogel‐based biomaterials show tremendous potential in diverse fields of biomedical engineering; however, one should also consider that not all aerogel‐based studies in the literature are indeed aerogels, considering the aerogel definition by the IUPAC. Therefore, this misconception about using aerogels in the literature constantly exists. To better classify the reviewed materials and avoid the misconception, in this article, we used the terminology aerogel‐inspired or aerogel‐like materials with some similarities in the synthesis and some materials properties like low‐density, the high extent of porosity to the aerogel systems. Even though aerogels and aerogel‐inspired materials demonstrated a significant breakthrough in some bio‐relevant applications, mainly drug delivery, still further research/development in other applications of regenerative medicine (i.e., wound healing and tissue engineering) is required. Moreover, aerogels applications for gene delivery and cell delivery have not yet been studied, leaving significant room for future research direction. As shown by researchers, the applicability of AM makes aerogel fabrication scalable and more promising for creating complex shapes and microstructures. This helps to engineer the porous structure with pore size and pore interconnectivity similar to the ECM as well as shaping them into customized shapes complying with the tissue defect site. However, still, further studies are required in this area to overcome the aerogels’ post‐synthesis shaping, expanding their applications for various biomedical applications and in particular paving their future clinical translation.

## Conflict of Interest

The authors declare no conflict of interest.
